# Moderately Flexible Copper–Triazolate Framework with Quinone-Based Linkers: Balancing Effective CO_2_ Capture and Low-Energy Regeneration

**DOI:** 10.1021/acs.inorgchem.6c02247

**Published:** 2026-08-06

**Authors:** Yao-Ting Wang, Tsu-Lien Hung, Anton S. Pozdeev, Alexander S. Ivanov, Hsin-Kuan Liu, Yu-Chun Chuang, Chung-Kai Chang, Jeng-Lung Chen, Ilja Popovs, Watchareeya Kaveevivitchai, Teng-Hao Chen

**Affiliations:** † Department of Chemical Engineering, 34912 National Cheng Kung University, Tainan City 70101, Taiwan; ‡ Center for Resilience and Intelligence on Sustainable Energy Research (RiSER), National Cheng Kung University, Tainan City 70101, Taiwan; § Core Facility Center, National Cheng Kung University, Tainan City 70101, Taiwan; ∥ Department of Chemical and Biomolecular Engineering, Vanderbilt University, Nashville, Tennessee 37235, United States; ⊥ Department of Nuclear Engineering, 4292 University of Tennessee, Knoxville, Tennessee 37996, United States; # 57815 National Synchrotron Radiation Research Center, Hsinchu City 300092, Taiwan; g Academy of Innovative Semiconductor and Sustainable Manufacturing, National Cheng Kung University, Tainan City 70101, Taiwan; h Institute of Clinical Pharmacy and Pharmaceutical Sciences, School of Pharmacy, College of Medicine, National Cheng Kung University, Tainan City 70101, Taiwan

## Abstract

The development of advanced physisorption-based adsorbents is essential for carbon dioxide (CO_2_) capture technologies. While metal–organic frameworks (MOFs) offer a promising alternative to traditional carbon capture methods, they often face a trade-off between high adsorption affinity and energy-intensive regeneration. In this work, we report an anionic copper–triazolate framework, NCKU-56 (NCKU = National Cheng Kung University), incorporating quinone-based organic linkers. The 1D channels exhibit moderate structural flexibility and reversible lattice adjustments that are specifically correlated with CO_2_ adsorption. This material demonstrates high CO_2_/N_2_ IAST selectivity of 65 and 315 (50/50, 15/85, v/v) at 298 K and a moderate isosteric heat of adsorption of 30.1 kJ mol^–1^, which facilitates rapid regeneration without high thermal energy penalties. Crystallographic analysis reveals that the selective uptake is driven by cooperative host–guest interactions, including quadrupole−π interactions (with quinone cores and triazolate rings) and hydrogen bonding between CO_2_ and [NH_2_(CH_3_)_2_]^+^ cations. Breakthrough experiments confirm the practical efficacy of NCKU-56 for CO_2_/N_2_ separation, highlighting the potential of integrating polar functional groups and π systems with moderate framework flexibility for energy-efficient carbon capture.

## Graphical abstract

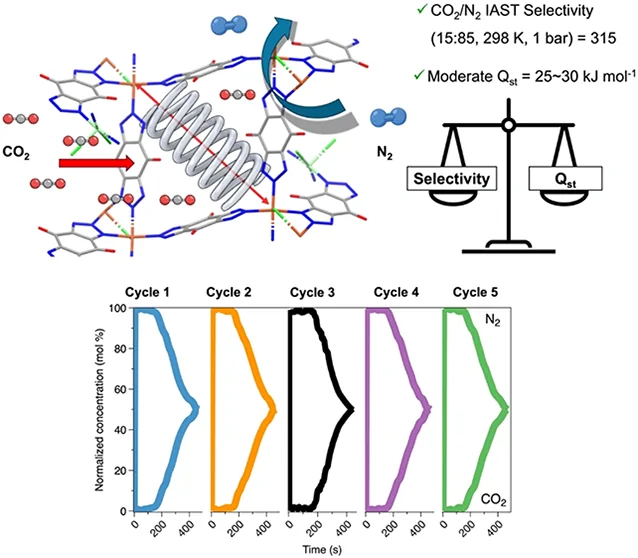

## Introduction

The continuous increase in atmospheric carbon dioxide (CO_2_), primarily driven by the combustion of fossil fuels and industrial emissions, is one of the most critical environmental and technological challenges of the 21st century. Global CO_2_ concentrations surpassed 426 ppm in 2025, and are directly correlated with rising global temperatures and the increasing frequency of extreme weather.^1–3^ Achieving carbon neutrality, therefore, demands the rapid development of scalable, energy-efficient technologies for CO_2_ capture and separation. Traditional methods, such as amine scrubbing, cryogenic distillation, and calcium looping, have significant trade-offs in terms of efficiency, cost, and sustainability.^4–6^ Amine-based absorption, though capable of high capacities, suffers from solvent degradation, corrosivity, and large thermal energy requirements for regeneration.^7,8^ Cryogenic separation yields high purity but requires intensive refrigeration.^9^ Calcium looping systems are limited by rapid sorbent deactivation due to sintering and attrition across multiple cycles.^10^ These constraints have motivated enduring searches for solid adsorbents capable of reversible CO_2_ capture through physical adsorption under mild conditions.

Metal–organic frameworks (MOFs), a type of crystalline porous material consisting of metal nodes and organic linkers, have fundamentally reshaped the design landscape of physisorbents,^11^ offering an promising alternative to conventional porous solids such as zeolites and activated carbons.^12,13^ For example, zeolites require significant amount of energy for thermal regeneration because of their strong affinity for CO_2_, and they also struggle with competitive water adsorption. Activated carbons lack the specific thermodynamic affinity and uniform pore sizes essential for effective CO_2_/N_2_ selectivity.^14,15^ In contrast, the structural modularity of MOFs enables the precise tailoring of both the pore architecture and the chemical environment to optimize gas–solid interactions,^16–18^ which can be achieved through the strategic selection of metal nodes and organic linkers.^19^ However, the design of these adsorbent materials remains constrained by an intrinsic trade-off between CO_2_ adsorption affinity and regeneration efficiency. MOFs featuring a high density of open metal sites, such as Mg-MOF-74, leverage strong Lewis acid–base interactions to achieve impressive CO_2_ capacities;^20,21^ yet, their high isosteric heats of adsorption (*Q*_st_ > 40 kJ mol^–1^) result in significant energy penalties during material regeneration. Conversely, chemically robust materials such as UiO-66 series^22,23^ or hydrophobic ZIF-8 ^24,25^ exhibit excellent structural integrity but suffer from limited selectivity (typically ∼ 20 or lower) due to weak electrostatic or quadrupolar interactions between their nonpolar pore environments and CO_2_ molecules. Consequently, the frontier of CO_2_ capture research focuses on identifying an optimal window of adsorption energy, typically 25–35 kJ mol^–1^, that ensures high CO_2_/N_2_ selectivity while maintaining the reversibility required for low-energy CO_2_ desorption.^26^ Achieving this delicate balance requires sophisticated strategies that move beyond static, rigid architectures; specifically, the synergistic coupling of multiple host–guest interactions with controlled structural flexibility is essential to enhance selective CO_2_ uptake and facilitate facile material regeneration.

Flexible MOFs exhibit a unique ability to undergo reversible structural transitions in response to specific guest molecules.^27–29^ Although archetypal flexible materials like MIL-53­(Al) show promise for molecular sieving, their inherent large-scale phase transitions often impose high energetic barriers, leading to sluggish adsorption kinetics or mechanical instability associated with drastic structural transformations during long-term cycling.^30^ Accordingly, there is an increasing shift toward developing frameworks with moderate flexibility, allowing subtle and fully reversible lattice adjustments.^31,32^ However, flexible frameworks lacking specific binding sites may undergo nonselective pore expansion driven purely by applied gas pressure, thereby accommodating competing gases and diluting overall separation efficiency.^33,34^ Integrating framework flexibility with targeted chemical recognition has the potential to address the trade-off between CO_2_ selectivity and the energy penalty of regeneration. Specifically, functionalizing pores with polar functional groups can induce localized electrostatic fields, significantly enhancing the thermodynamic affinity for CO_2_ through targeted dipole–quadrupole interactions.^35,36^ In addition, the binding energy of CO_2_-π interaction is usually high enough to selectively capture CO_2_, but low enough that the CO_2_ can be released by a slight change in pressure or temperature.^37^ In this regard, quinone-based molecules can be perfect platforms for providing polar carbonyl groups and a polarized π-system in a compact structure.^37–39^ However, the development of flexible MOFs that incorporate quinone-based moieties remains largely unexplored.^40^

In this work, 2H,6H-benzo­[1,2-*d*]­[4,5-*d*′]­bistriazolequinone (H_2_BBTQ) is chosen as the organic linker with terminal triazolate groups that can coordinate with metal nodes and also provide additional conjugated systems. H_2_BBTQ has a compact quinone-cored molecular structure, enabling the formation of a polar and microporous environment with π systems. By reacting H_2_BBTQ with Cu^2+^, we report NCKU-56 (NCKU = National Cheng Kung University), [Cu_2_(BBTQ)_2.5_Cl_2_]^3–^·3­[NH_2_(CH_3_)_2_]^+^, an anionic copper–triazolate framework that exhibits moderate and reversible elasticity. This material exhibits lattice adjustments correlated with CO_2_ adsorption, enabling high CO_2_ selectivity and a moderate Q_st_ for rapid regeneration. Through comprehensive crystallographic and gas-sorption analyses, we establish the molecular origin of its responsive behavior. We provide a detailed discussion on the structure-performance relationship of NCKU-56, demonstrating how its flexibility and multiple CO_2_-framework interactions balance selectivity, kinetics, and low-energy regeneration for CO_2_ separation.

## Experimental Section

### Synthesis of NCKU-56

H_2_BBTQ (60 mg, 0.40 mmol)^41^ and CuCl_2_·2.5H_2_O (100 mg, 0.60 mmol) were dissolved in 40 mL of *N*,*N*-dimethylformamide (DMF) and 10 mL of isopropanol using an ultrasonic bath to ensure complete dissolution. The solution was then heated at 60 °C for 7 d in a sealed glass vial. After cooling to room temperature, dark green single crystals were obtained. The crystals were washed with 50 mL of DMF and 50 mL of ethanol, then filtered. The final product was activated using supercritical CO_2_ (scCO_2_) drying (see Supporting Information for details). The yield calculated from the activated sample was 80% based on H_2_BBTQ. FT-IR: 1696 (C = O), 1680 (C = O), 1651 (C = N), and 1446 (C–N) cm^–1^. Elemental analysis calculated for activated NCKU-56 (C_21_H_24_Cl_2_Cu_2_N_18_O_5_): C, 29.2; H, 4.2; N, 26.9. Found: C, 29.3; H, 3.8; N, 27.1. CCDC 2545590 and 2545591 contain the supplementary crystallographic data for the as-synthesized and scCO_2_-activated NCKU-56, respectively. These data can be obtained free of charge from the Cambridge Crystallographic Data Centre via www.ccdc.cam.ac.uk/data_request/cif

### Gas Sorption Measurements

Gas sorption isotherms were measured using a Micromeritics 3Flex high-performance gas adsorption analyzer. Approximately 100 mg of the scCO_2_-activated sample was degassed under vacuum at 343 K for 12 h before measurement. Each sample tube was capped, evacuated to 1 × 10^–5^ Torr, and heated to 323 K for 4 h. Isotherms were collected at different temperatures over a pressure range of 0–760 mmHg. High-purity gases, including N_2_ (99.9995%), CO_2_ (99.99%), and He (99.9995%), were utilized for free-space corrections and gas sorption experiments. The *Q*_st_ was calculated from CO_2_ adsorption isotherms collected at 273, 298, and 313 K.

### Single-crystal X-ray Diffraction Analysis upon CO_2_ Adsorption

A single crystal of NCKU-56 (ca. 80 × 60 × 100 μm^3^) was mounted in a capillary tube. The crystal was transferred into a customized gas-loading cell holder (Figure S1). The sample holder was evacuated to <1 × 10^–3^ Torr, followed by dosing with CO_2_ (99.999%) at 1 bar and 298 K. The gas pressure was maintained for 3 h to ensure an adsorption equilibrium. Single-crystal X-ray diffraction (SCXRD) data were collected at the CO_2_-loaded condition at 100 K on a Bruker D8 VENTURE diffractometer using Cu Kα radiation (λ = 1.5418 Å). CCDC 2545592 contains the supplementary crystallographic data for CO_2_-loaded NCKU-56. These data can be obtained free of charge from the Cambridge Crystallographic Data Centre via www.ccdc.cam.ac.uk/data_request/cif

### Kinetic Study of CO_2_ Adsorption

Kinetic CO_2_ adsorption measurements were performed using a Mettler Toledo TGA 2 thermogravimetric analyzer. A sample of approximately 3.9 mg was loaded into a standard alumina crucible and heated to 343 K under a constant N_2_ flow of 20 cm^3^ min^–1^ for 10 min to remove moisture and contaminants. Following this pretreatment, the system was cooled to 298 K and held for 30 min under a continuous N_2_ purge to ensure thermal and weight stabilization. The gas feed was then switched to pure CO_2_ at flow rates of either 15 or 30 cm^3^ min^–1^, and the resulting weight gain, corresponding to CO_2_ uptake, was recorded over an adsorption period of 100 min.

### *In Situ* Synchrotron Powder X-ray Diffraction Analysis upon Gas Adsorption

*In situ* powder X-ray diffraction (PXRD) experiments of NCKU-56 under CO_2_ and N_2_ adsorption were conducted at the Taiwan Photon Source (TPS) 19A beamline at the National Synchrotron Radiation Research Center (NSRRC), Hsinchu, Taiwan. The wavelength of the incident X-rays was 0.82656 Å (15 keV), delivered from an in-vacuum undulator (IU22). The PXRD patterns were recorded using a MYTHEN 24K position-sensitive detector. The powder sample was densely packed into a borosilicate glass capillary tube with a 0.7 mm diameter. The entire system was outgassed at 1 × 10^–3^ bar and 298 K for 30 min before gas adsorption experiments. CO_2_ and N_2_ were then introduced into the capillary tube at 298 K. PXRD patterns were recorded at 3 min intervals with an exposure time of 30 s.

### Breakthrough Experiments

Dynamic gas separation measurements were performed using a PSA-300LC system (L&C Science and Technology, Hialeah, FL, USA). Approximately 1.38 g of NCKU-56 was packed into a stainless-steel column (4 × 150 mm). The packed column was activated at 323 K under vacuum for 4 h before measurements. Before each run, the column was purged with He (10 cm^3^ min^–1^) at 298 K for 5 min. A binary gas mixture of CO_2_/N_2_ (50/50, v/v) was introduced at flow rates of 3 and 5 cm^3^ min^–1^ and pressures of 1 and 2 bar. The effluent composition was analyzed continuously using a mass spectrometer (Extorr XT-100). For cyclic breakthrough experiments, the adsorbent was regenerated under vacuum at 298 K for 1 h before the next run.

## Results and Discussion

NCKU-56 was synthesized via solvothermal reaction, yielding dark-green crystals. SCXRD analysis reveals that NCKU-56 crystallizes in a three-dimensional (3D) coordination network (Figure 1A). This connectivity forms one-dimensional (1D) channels of approximately 6–7 Å wide, aligned along the crystallographic *c*-axis. The framework is constructed from mononuclear Cu^2+^ centers, interconnected by deprotonated BBTQ linkers and bridging chloride anions (Figure 1B). The asymmetric copper nodes show two distorted square-pyramidal coordination geometries, denoted as Cu1 and Cu2. Specifically, the Cu1 site is coordinated by four triazolate N atoms and one apical Cl^–^. The Cu2 site is coordinated by three triazolate N atoms and two Cl^–^. The asymmetric topology of the copper-triazolate network suggests a considerable potential for structural deformation. The negatively charged [Cu_2_(BBTQ)_2.5_Cl_2_]^3–^ network is balanced by [NH_2_(CH_3_)_2_]^+^ cations, from the decomposition of DMF molecules (Figure S2).^42^ These cations also function as molecular pillars to enhance the structural stability.

**Figure 1:**
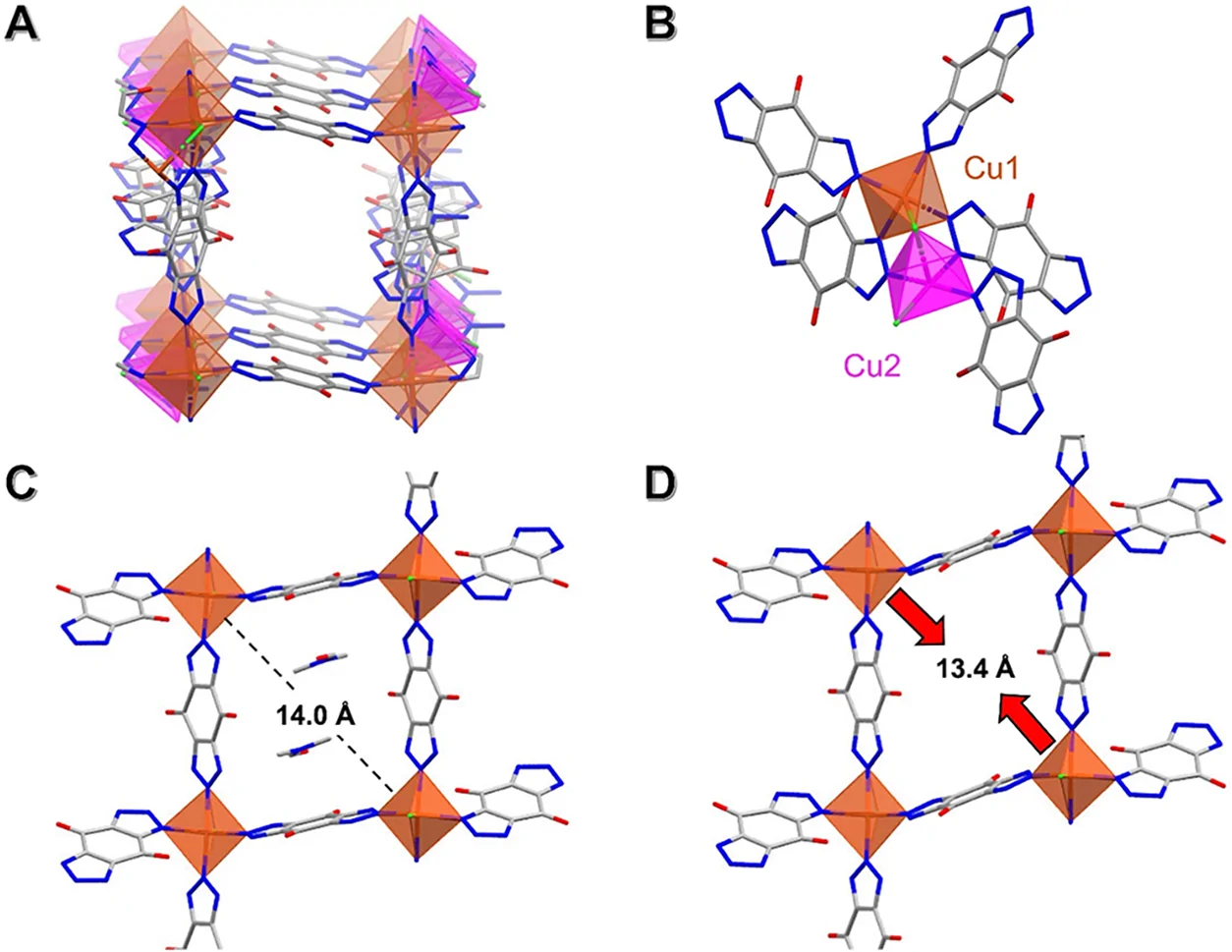
(A) The crystal structure of the 1D channel of as-synthesized NCKU-56 viewed along the *c-*axis. Solvent molecules and [NH_2_(CH_3_)_2_]^+^ cations are omitted for clarity. (B) Two Cu coordination symmetries of NCKU-56. (C) The DMF-loaded pore structure of as-synthesized NCKU-56. (D) The pore structure of scCO_2_-activated NCKU-56. [NH_2_(CH_3_)_2_]^+^ cations are omitted for clarity. Element colors: C—gray, O—red, N—blue, Cl—green, and Cu—brown and magenta.

To confirm the oxidation state of the metal nodes, Cu K-edge X-ray absorption near-edge structure (XANES) spectra were collected (Figure S6). The edge position of NCKU-56 aligns well with the CuO reference, confirming the exclusive presence of divalent copper. Furthermore, the chemical stability of the MOF was assessed by immersing the crystals in common organic solvents for 24 h (Figure S7). The PXRD patterns of all solvent-treated samples remain consistent with the simulated pattern. The chemical environment of the quinone-functionalized pores was investigated using Fourier-transform infrared (FT-IR) spectroscopy. The free H_2_BBTQ ligand exhibits sharp and intense ν­(C = O) stretching vibrations at 1700 and 1715 cm^–1^, which are characteristic of quinoid carbonyl groups. Upon the formation of NCKU-56, this characteristic band shifts to 1680 and 1696 cm^–1^, indicating the enhanced conjugation of π systems (Figure S8).

In the as-synthesized phase, the pores of NCKU-56 are occupied by DMF molecules and [NH_2_(CH_3_)_2_]^+^ cations (Figure S2). Upon activation with scCO_2_, the DMF molecules are evacuated while the [NH_2_(CH_3_)_2_]^+^ cations remain confined within the 1D channels. SCXRD analysis of the activated sample reveals a noticeable lattice contraction, with the unit cell volume decreasing from 4316 to 4021 Å^3^ (−6.8%). A scissoring motion at the metal nodes accommodates this small volume reduction. This dynamic adjustment is clearly evidenced by the decrease in the cross-channel Cu···Cu diagonal distance from 14.0 Å in the as-synthesized phase (Figure 1C) to 13.4 Å in the activated phase (Figure 1D). This structural response is characterized primarily by the compression along the *a*- and *b*-axes. The moderate volume change in NCKU-56 allows the removal of guest molecules without structural collapse, possibly owing to the supporting [NH_2_(CH_3_)_2_]^+^ cations. The activation efficiency was verified by TGA. The as-synthesized material displays a 10% weight loss up to 150 °C, corresponding to the release of trapped DMF molecules (Figure S9). The scCO_2_-activated NCKU-56 exhibits approximately 8% weight loss below 60 °C, which can be attributed to gas desorption (Figure S10). This phenomenon indicates that this material can adsorb gases in the ambient environment.

The porosity of the activated NCKU-56 was probed by N_2_ adsorption–desorption measurements at 77 K (Figure S11). The isotherm exhibits a negligible uptake at low pressures, followed by a sudden increase near *P*/*P*_o_ = 0.06, indicative of a pressure-induced structural transition. A pronounced hysteresis is observed during desorption, suggesting that the structural closure and reopening are not synchronized under cryogenic conditions. In contrast, the CO_2_ adsorption measurement at 195 K shows a typical type-I isotherm, indicating facile access of CO_2_ molecules to the channels of NCKU-56 (Figure S12). Using the cross-sectional area of CO_2_ (0.170 nm^2^), the Brunauer–Emmett–Teller (BET) surface area of NCKU-56 is determined as 192.5 m^2^ g^–1^ from the 195 K CO_2_ adsorption isotherm (Figure S13 and Table S4). Importantly, this pressure-induced gate-opening for gas adsorption is completely suppressed at ambient temperature (Figure 2A). Gas adsorption isotherms of NCKU-56 at 298 K reveal a selective CO_2_ adsorption over N_2_. No stepwise adsorption is observed, indicating that the gate-opening behavior is minimized in this moderately flexible structure. At 1 bar, the CO_2_ uptake reaches 24.8 cm^3^ g^–1^, whereas N_2_ adsorption remains negligible below 1 cm^3^ g^–1^. Since the channels are not closed, the exclusion of N_2_ is very likely due to the polar carbonyl groups.^43^ The polar and ionic framework also readily adsorbs water vapor (Figure S14). However, after exposure to humidity, NCKU-56 can be reactivated, and its CO_2_ uptake remains close to that of the scCO_2r_-activated sample (Figure S15). Powder X-ray diffraction (PXRD) analysis shows the high crystallinity of NCKU-56 after moisture exposure and gas adsorption experiments, confirming its structural stability (Figure S16).

**Figure 2:**
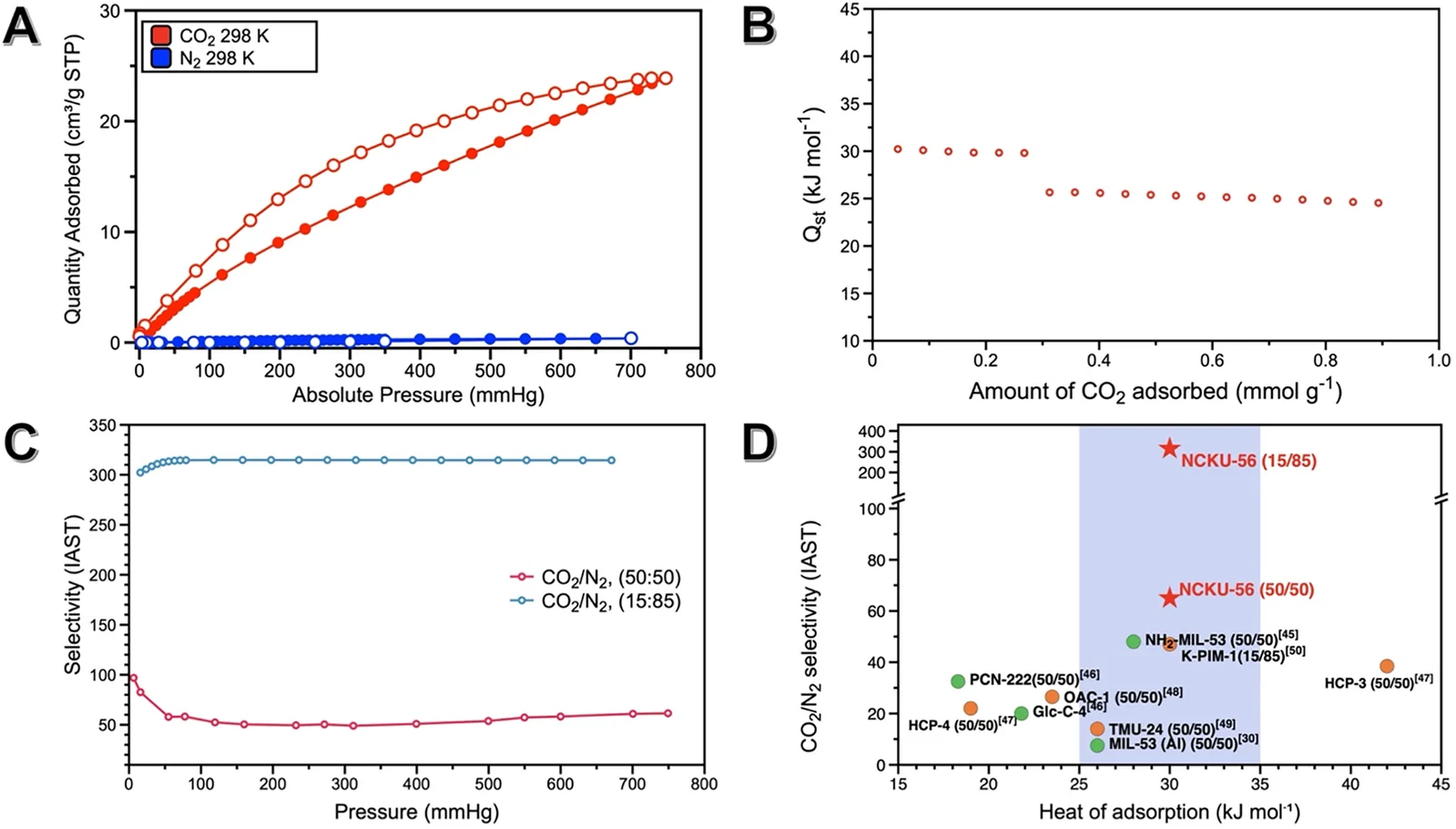
(A) CO_2_ and N_2_ sorption isotherms of NCKU-56 at 298 K. (B) *Q*_st_ of NCKU-56 for CO_2_ adsorption. (C) IAST selectivity of NCKU-56 toward gas mixtures CO_2_/N_2_ (50/50 and 15/85, v/v) at 298 K. (D) Comparison of CO_2_/N_2_ selectivity and *Q*_st_ of CO_2_ adsorption for NCKU-56, quinone/carbonyl-based porous materials (orange), and flexible MOFs (green) at ambient conditions.^30,45–50^

To evaluate the CO_2_-framework interactions, the *Q*_st_ of CO_2_ adsorption is calculated from the CO_2_ adsorption isotherms at 273, 298, and 313 K (Figures 2B and S17). The Q_st_ value at near-zero coverage is 30.1 kJ mol^–1^, within the range of π-based physisorption (20–35 kJ mol^–1^). As the CO_2_ loading increases, the *Q*_st_ profile remains stable. This result suggests a high degree of energetic homogeneity across the adsorption sites.^44^ This discrimination between CO_2_ and N_2_ uptake is attributed to the combination of confined pore size, a polar environment, and moderate CO_2_-framework interactions.

The ideal adsorbed solution theory (IAST) selectivity of NCKU-56 for CO_2_/N_2_ separation is calculated from the single-component CO_2_ and N_2_ adsorption isotherms at 298 K. The experimental data are fitted using the dual-site Langmuir–Freundlich (DSLF) model (Figure S19), which accounts for the energetic heterogeneity of the pore environment. Together with the flat Q_st_ curve shown in Figure 2B, this suggests that the heterogeneous adsorption sites have very similar affinities.^51^ Based on the derived DSLF parameters, IAST calculations are performed for a CO_2_/N_2_ mixture (50:50, v/v), revealing a high selectivity of 65 (Figure 2C). To further evaluate the feasibility of NCKU-56 for industrial flue gas purification, the predicted CO_2_/N_2_ IAST selectivity of a binary CO_2_/N_2_ (15/85, v/v) mixture reaches an outstanding value of 315. While NCKU-56 exhibits a moderate static CO_2_ adsorption capacity, its prominent selectivity underscores its competence for efficient carbon capture from diluted postcombustion streams.

For energy-efficient CO_2_ separation based on physisorption, an ideal solid sorbent must exhibit high selectivity to ensure product purity while maintaining a moderate Q_st_ to minimize the thermal energy penalty during regeneration. Despite their high working capacity, flexible MOFs with large structural expansion often require high pressure to open their pores, thereby increasing energy consumption. Thus, in Figure 2D, we compare NCKU-56 with quinone/carbonyl-based porous materials and flexible MOFs in terms of IAST selectivity and *Q*_st_ calculated for CO_2_/N_2_ separation at ambient temperatures and pressures. The trade-off of NCKU-56 between CO_2_/N_2_ selectivity and *Q*_st_ of CO_2_ adsorption (25–35 kJ mol^–1^) outperforms other materials. For example, the carbonyl-functionalized microporous polymers (e.g., HCP-3 and HCP-4)^47^ with rigid structures exhibit adsorption enthalpies either higher (*Q*_st_ > 40 kJ mol^–1^) or lower (*Q*_st_ < 20 kJ mol^–1^) than the desired range. The flexible NH_2_-MIL-53 ^52^ with polar amino groups exhibits significantly enhanced selectivity and *Q*_st_ compared to the archetypal breathing MOF, MIL-53­(Al).^53^ Conversely, while introducing structural flexibility can dynamically enhance gas discrimination, frameworks that undergo large-scale phase transitions frequently exhibit pronounced hysteresis and phase coexistence during gas uptake.^31^ These behaviors not only compromise CO_2_/N_2_ selectivity under practical mixed-gas conditions but also lead to structural fatigue and sluggish kinetics during repeated cycling, complicating their deployment in continuous-flow operations. Therefore, the combination of polar environment, π systems, and a moderately flexible framework in NCKU-56 provides a unique platform for effective CO_2_ capture and low-energy regeneration.

To elucidate the host–guest interactions responsible for selective CO_2_ uptake, we performed SCXRD analysis of NCKU-56 upon CO_2_ loading. A single crystal of activated NCKU-56 was mounted in a customized gas-loading cell holder, evacuated, and then dosed with CO_2_ at 1 bar and 298 K. The SCXRD data were collected at 100 K. The lattice of the CO_2_-loaded NCKU-56 exhibits a 14.3% volume contraction (from 4021 to 3443 Å^3^) compared to that of the scCO_2_-activated phase, as well as the decreased cross-channel Cu···Cu diagonal distance of 12.4 Å (Figure 3A). The CO_2_-loaded structure reveals localized electron density within the 1D channels, corresponding to oriented CO_2_ molecules situated near the charge-compensating [NH_2_(CH_3_)_2_]^+^ cations, the triazolate rings, and the quinone cores (Figure 3B). A detailed crystallographic analysis reveals three distinct, cooperative interactions responsible for the lattice contraction, thereby enabling more efficient host–guest interactions. First, the electropositive carbon atom of the CO_2_ molecule engages in a favorable electrostatic interaction with the electron-rich π system of the five-membered triazolate rings, at a distance of 3.1 Å. Second, the CO_2_ molecules interact with the polarized π system of quinone cores, at a distance of 3.1 Å from the ring centroids. Third, the electronegative oxygen atoms of the CO_2_ guest act as hydrogen-bond acceptors, forming short contacts (2.6 Å) with the N–H groups of the [NH_2_(CH_3_)_2_]^+^ cations. These supramolecular weak interactions cooperatively stabilize the entrapped CO_2_ molecules, aligning well with the results mentioned above, which show that the heterogeneous adsorption sites have very similar affinities. These thermodynamic factors, derived from crystallographic insights, account for the selective CO_2_ uptake observed in the gas adsorption measurements, and confirm that the framework is flexible under CO_2_ loading at ambient conditions.

**Figure 3:**
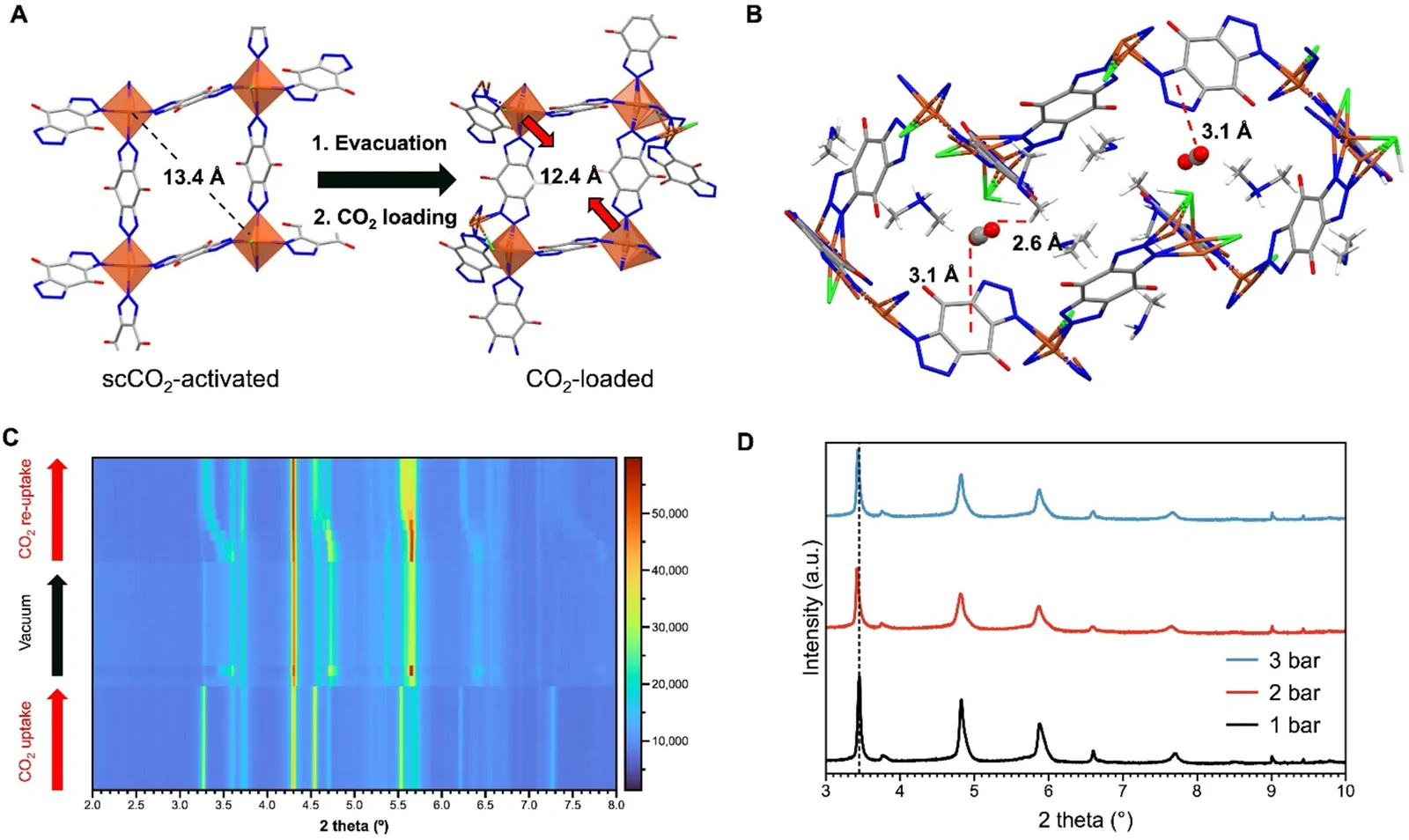
(A) The contraction of NCKU-56 lattice upon CO_2_ loading via SCXRD analysis. [NH_2_(CH_3_)_2_]^+^ cations and CO_2_ molecules are omitted for clarity. (B) Crystal structure of NCKU-56 loaded with CO_2_ molecules. (C) *In situ* synchrotron PXRD analysis of NCKU-56 under CO_2_ adsorption/desorption process with data collected every 3 min at 298 K. (D) *In situ* synchrotron PXRD patterns of NCKU-56 loading with CO_2_ at 1, 2, and 3 bar.

To bridge the localized host–guest interactions observed in the static single-crystal structure with the dynamic behavior of the bulk materials, *in situ* synchrotron PXRD measurements were performed under alternating CO_2_ and vacuum atmospheres at 298 K (Figure 3C). During the vacuum process, the primary diffraction peaks shift to higher 2θ values, indicating an 8% lattice contraction. The framework undergoes a drastic unidirectional compression along the crystallographic *b*-axis, shrinking from 27.1 to 23.3 Å, while the *a*- and *c*-axes remain consistent. This one-dimensional flexibility allows the framework to dynamically and reversibly adjust its pore volume to accommodate guest molecules without compromising its overall topological integrity. Crucially, when the system is reloaded with CO_2_, these peaks return gradually to their original positions, demonstrating a fully reversible structural transformation between the contracted and expanded states. Furthermore, the absence of new diffraction peaks or significant intensity loss during cycling confirms that the guest-induced lattice adjustment occurs via a continuous and elastic transition rather than a reconstructive phase change. In contrast, the *in situ* synchrotron PXRD patterns of NCKU-56 are identical over the N_2_ adsorption–desorption processes (Figure S20). Many flexible MOFs exhibit complex *Q*_st_ steps due to the high energy penalty associated with structural opening. In this regard, the guest-induced lattice adjustment of NCKU-56 is energetically subtle.^54,55^ This “low-barrier” responsiveness enables the framework to operate within a reversible elastic regime, where host–guest interactions provide sufficient driving force for pore expansion while allowing seamless regeneration upon depressurization. At higher pressures of 2 and 3 bar (Figure 3D), the first characteristic peak of CO_2_-loaded NCKU-56 shifts 0.03° to the lower 2θ values, indicating the slight expansion of the structure, which might allow the accommodation of more gas molecules.

A comparison of the density functional theory (DFT)-optimized empty and CO_2_-loaded frameworks (Table S6) reveals a strongly anisotropic contraction: the *b*-axis shortens by 12.5% and the monoclinic β angle opens by 2.3°, while the *a*-axis is essentially unchanged. Crucially, the primary coordination bonds are rigid with the Cu–Cl (2.43 Å) and Cu–N (≈2.03 Å) bond lengths being identical in both phases. The framework therefore accommodates a ∼15% volume reduction without any compression of the Cu–Cl or Cu–N bonds. The deformation is purely angular, arising from a hinging (scissoring) reorientation of the linkers about the rigid copper nodes together with a monoclinic shear. This low-energy angular mechanism, involving no bond compression or bond rupture, accounts for the moderate, fully reversible, and low-barrier flexibility of NCKU-56. To further quantify these interactions, the CO_2_ and N_2_ adsorption energies were computed by periodic DFT (see Supporting Information for details). The computed CO_2_ adsorption energy (−33.6 kJ mol-1) is consistent with the experimental *Q*_st_ (30.1 kJ mol-1), and the optimized geometry shows CO_2_ bound at the triazolate rings through its electropositive carbon (C···N ≈ 3.0 Å) while remaining essentially linear, in line with weak, nonactivating physisorption (Figure 4, Table S7). By contrast, N_2_ interacts more weakly (−23.9 kJ mol-1) and does not localize at any specific site, providing an atomistic rationale for the observed CO_2_/N_2_ selectivity.

**Figure 4:**
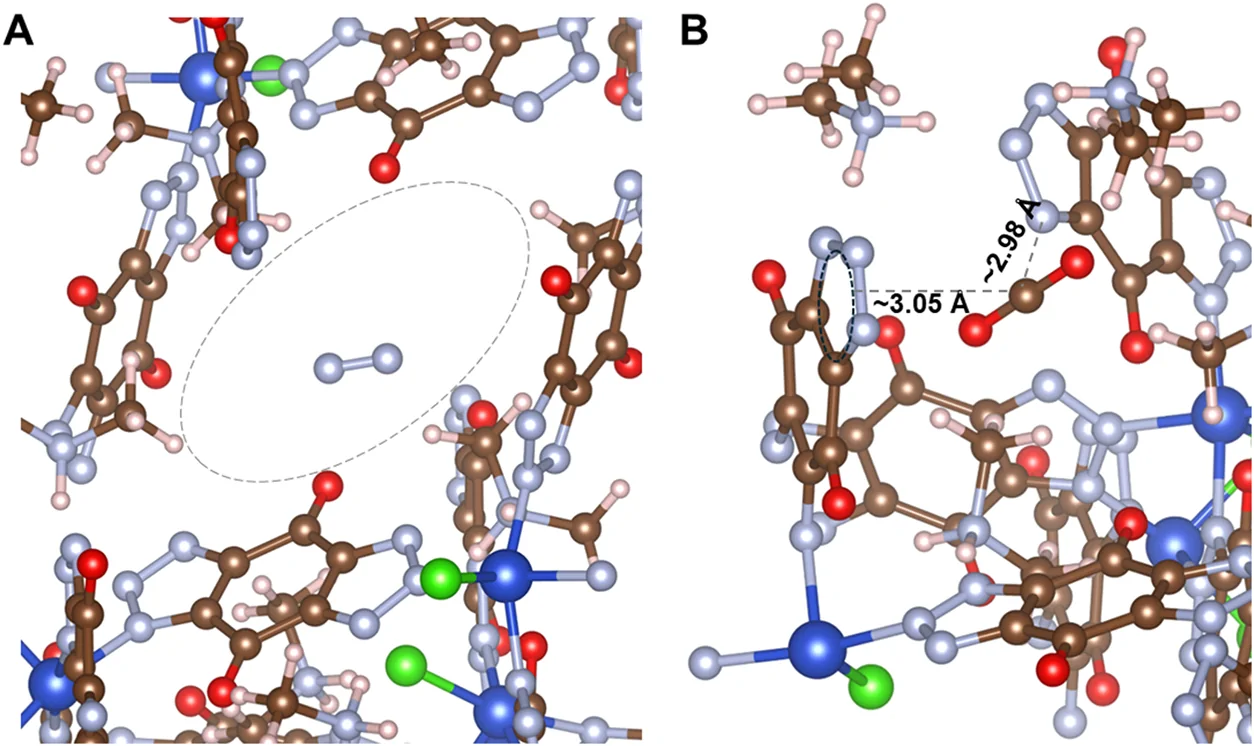
DFT-optimized geometries of (A) N_2_ and (B) CO_2_ in the channel of NCKU-56.

Beyond thermodynamic factors, rapid adsorption kinetics are a critical prerequisite for the practical deployment of porous materials in dynamic gas separations. To evaluate the adsorption kinetics of NCKU-56, kinetic CO_2_ uptake profiles were recorded using a TGA (Figure 5). Following thermal activation and baseline stabilization under a continuous N_2_ purge at 298 K, the gas feed was abruptly switched to pure CO_2_ to simulate a dynamic adsorption environment. The material exhibits a rapid kinetics, attaining over 80% of its equilibrium CO_2_ capacity within 20 min at 1 bar. The CO_2_ uptake amount (about 7.5 wt %) perfectly matches the weight loss of scCO_2_-activated NCKU-56 during TGA analysis (Figure S12), confirming that the trapped gas under ambient conditions is CO_2_. When the CO_2_ feed flow rate is doubled from 15 to 30 cm^3^ min^–1^, the adsorption capacity slightly decreases from 7.03 to 6.78 wt % due to the higher flow rate, but the initial rate of weight gain increases from 0.17 to 0.22 wt % min^–1^. This positive dependence on the feed rate indicates that the adsorption rates are primarily governed by external mass transfer across the gas–solid boundary layer rather than by sluggish intracrystalline diffusion.^56^ Consequently, this result highlights the low internal diffusion resistance of NCKU-56, demonstrating that the 1D channels are fully accessible and unobstructed for CO_2_. From a process engineering perspective, this feature is highly advantageous, as it ensures rapid transport and full utilization of the active sites within an adsorbent bed under continuous-flow conditions.

**Figure 5:**
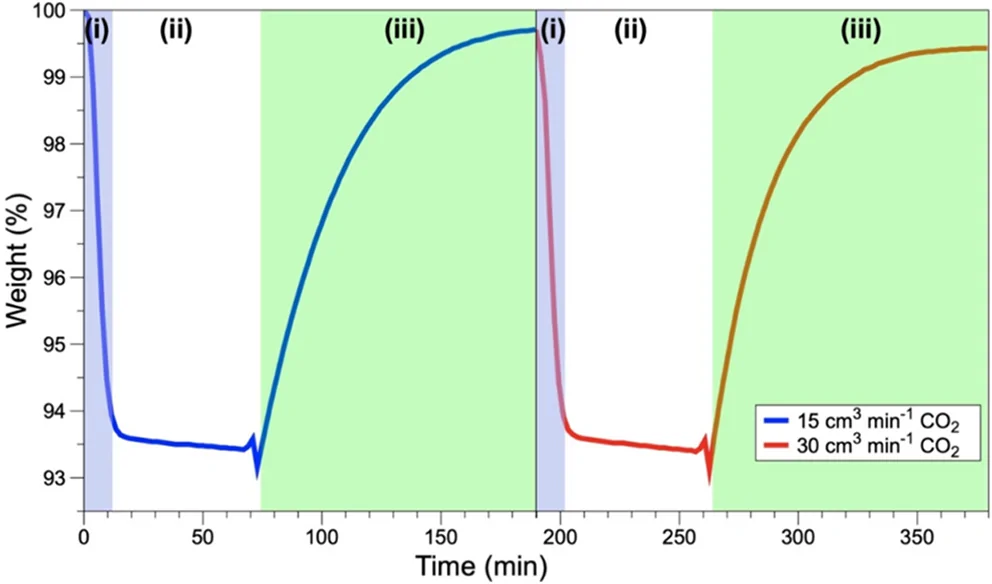
Kinetic CO_2_ adsorption profiles of NCKU-56 using TGA. The experimental procedures: (i) activation at 343 K with a N_2_ flow rate of 20 cm^3^ min^–1^ for 10 min; (ii) cooling to 298 K with a N_2_ flow rate of 20 cm^3^ min^–1^ for 30 min; (iii) CO_2_ adsorption at 298 K and a flow rate of 15 (blue) or 30 (red) cm^3^ min^–1^ for 100 min.

To clarify the fundamental role of this moderate flexibility, the retained description of moderate flexibility represents a dynamic gating mechanism governed by an energy barrier. Under cryogenic conditions, guest molecules can overcome this barrier to induce structural opening, as evidenced by the stepwise N_2_ sorption at 77 K (Figure S11). However, at ambient temperature (298 K), where thermal energy alone is insufficient to trigger nonspecific lattice expansion, an incoming guest must possess a strong specific affinity to overcome the deformation barrier. The smaller kinetic diameter (3.30 Å) and strong quadrupole moment of CO_2_ enable favorable host–guest interactions with the polar quinone and triazolate moieties, smoothly triggering a localized, reversible expansion (as confirmed by the CO_2_-loaded SCXRD study) that opens the pore apertures. In contrast, N_2_ (3.64 Å) lacks sufficient affinity to trigger this localized structural adaptation at 298 K. Without this flexible gating barrier, N_2_ might otherwise exhibit nonspecific physical uptake into the channels; instead, the channels remain strictly closed to N_2_ at ambient conditions (uptake <0.36 cm^3^ g^–1^ at 1 bar). This flexibility-driven gate control directly accounts for the remarkable CO_2_/N_2_ IAST selectivity (315 for 15/85, v/v). Furthermore, because this gating behavior is kinetically governed and highly reversible without trapping guests in deep thermodynamic wells, NCKU-56 achieves full CO_2_ desorption under ambient conditions within 1 h, providing a fundamental structural basis for balancing high selectivity with low-energy regeneration.

To further validate this separation efficiency under dynamic flow conditions, column breakthrough experiments were conducted using an equimolar CO_2_/N_2_ binary mixture at 298 K, flow rates of 3 and 5 cm^3^ min^–1^, and pressures of 1 and 2 bar, respectively (Figure 6A). Upon introducing the gas mixture, N_2_ eluted immediately at the column outlet with steep breakthrough curves. These profiles demonstrate that NCKU-56 can effectively capture CO_2_ under dynamic flow conditions. The calculated CO_2_ uptake capacities at 1 and 2 bar are 8.0 and 11.5 cm^3^ g^–1^, respectively. Along with its structural expansibility, NCKU-56 is promising for high-pressure separation technologies. Most importantly, the retained CO_2_ could be completely desorbed under vacuum at 298 K within 1 h, showing the facile regeneration without thermal input. Furthermore, five consecutive adsorption–desorption cycles were performed at a flow rate of 5 cm^3^ min^–1^, 298 K, and 2 bar, and no significant degradation in breakthrough time or in the shape of the elution profile was observed (Figure 6B). To verify the thermodynamic reversibility and capacity retention of the recycled adsorbent, a full static CO_2_ adsorption–desorption isotherm was remeasured immediately following the fifth cycling process, which perfectly replicated the pristine sorption profile without any degradation (Figure S21). Concurrently, the structural integrity of the cycled material was further confirmed by PXRD analysis, which showed no obvious changes compared to the pristine sample (Figure S22). These results are in good agreement with the high CO_2_/N_2_ IAST selectivity, the moderate Q_st_ of CO_2_ adsorption from static gas adsorption measurements, and the fast adsorption kinetics from TGA experiments.

**Figure 6:**
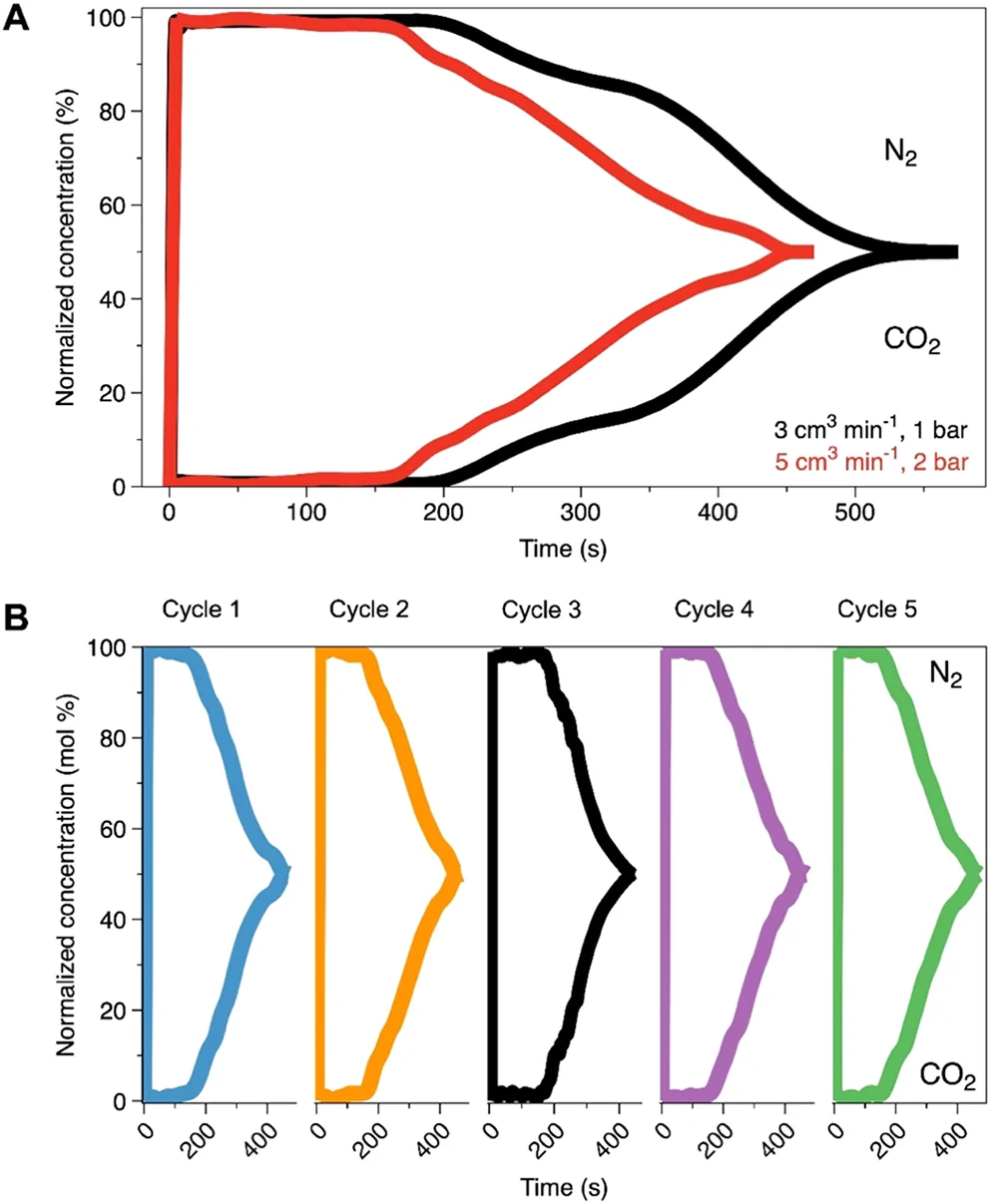
(A) Breakthrough curves (CO_2_/N_2_, 50/50, v/v) of NCKU-56 at 298 K. (B) Five repeated breakthrough curves (CO_2_/N_2_, 50/50, v/v) of NCKU-56 at a flow rate of 5 cm^3^ min^–1^, 298 K, and 2 bar.

## Conclusion

In summary, this study demonstrates that NCKU-56 successfully bridges the gap between high-selectivity gas discrimination and low-energy regeneration for carbon capture. By incorporating quinone-based moieties into a moderately flexible copper–triazolate framework, this material selectively captures CO_2_ with fast kinetics at ambient conditions, and also can be easily regenerated at 298 K. The combination of a polar pore environment, π systems, and subtle lattice responsiveness allows NCKU-56 to outperform many reported quinone/carbonyl-based and flexible adsorbents in the trade-off between high CO_2_/N_2_ IAST selectivity of 65 and 315 (50/50, 15/85, v/v) at 298 K and a moderate *Q*_st_ of 30.1 kJ mol^–1^. SCXRD and *in situ* synchrotron PXRD confirm that the framework undergoes a fully reversible, elastic transition during CO_2_ adsorption and desorption cycles, maintaining structural integrity without the high-pressure gate-opening, structural fatigue, or sluggish kinetics typical of large-scale breathing MOFs. These results provide a strategic design principle for the next generation of porous materials aimed at practical, large-scale, and energy-efficient carbon capture and industrial gas separation.

## Supplementary Material



## Notes

Detailed experimental methods and material characterization; synthesis protocols for the organic ligands H_2_BBTQ and NCKU-56; procedures for scCO_2_ activation; calculations for DSLF modeling, *Q*_st_, and IAST selectivity; XANES spectra, TGA profiles, FT-IR spectra, and PXRD patterns; customized setup for SCXRD analysis upon CO_2_ adsorption; crystallographic data tables for the as-synthesized, scCO_2_-activated, and CO_2_-loaded NCKU-56 (PDF)
