# Supplementary material for: Moderately Flexible Copper–Triazolate Framework with Quinone-Based Linkers: Balancing Effective CO2 Capture and Low-Energy Regeneration
Source: Inorg Chem. 2026 Aug 6;65(32):18738–47. doi: 10.1021/acs.inorgchem.6c02247 (PMC13488403; doi:10.1021/acs.inorgchem.6c02247)
Supplement: Supplementary file 1 [file ic6c02247_si_001.pdf]

Supporting Information

A Moderately Flexible Copper–Triazolate Framework with Quinone-based Linkers: Balancing Effective CO_2 Capture and Low-energy Regeneration

Yao-Ting Wang,[a,b] Tsu-Lien Hung,[c] Anton S. Pozdeev,[d] Alexander S. Ivanov,[e] Hsin-Kuan Liu,[c] Yu-Chun Chuang,[f] Chung-Kai Chang,[f] Jeng-Lung Chen,[f] Ilja Popovs,[e] Watchareeya Kaveevivitchai,[a,b,g]* and Teng-Hao Chen[h]*

[a] Department of Chemical Engineering, National Cheng Kung University, Tainan City 70101, Taiwan

[b] Center for Resilience and Intelligence on Sustainable Energy Research (RiSER), National Cheng Kung University, Tainan City 70101, Taiwan

[c] Core Facility Center, National Cheng Kung University, Tainan City 70101, Taiwan

[d] Department of Chemical and Biomolecular Engineering, Vanderbilt University, Nashville, TN 37235, United States.

[e] Department of Nuclear Engineering, University of Tennessee, TN 37996, United States

[f] National Synchrotron Radiation Research Center, Hsinchu City 300092, Taiwan

[g] Academy of Innovative Semiconductor and Sustainable Manufacturing, National Cheng Kung University, Tainan City 70101, Taiwan

[h] Institute of Clinical Pharmacy and Pharmaceutical Sciences, School of Pharmacy, College of Medicine, National Cheng Kung University, Tainan City 70101, Taiwan

General Methods and Materials

All starting materials and solvents were obtained from commercial suppliers (Sigma-Aldrich and TCI) and used without further purification. Powder X-ray diffraction (PXRD) patterns were collected on a Bruker D8 Advance diffractometer using Cu Kα radiation (λ = 1.5406 Å). Ultrahigh-purity gases N_2 (99.9995%), CO_2 (99.999%), and He (99.9995%) were purchased from Yun Shan, Ltd. (Taiwan). Thermogravimetric analysis (TGA) was performed on a Mettler Toledo TGA 2 Star System under N_2 flow, typically from 25 to 800 °C at a heating rate of 5 °C min^{-1}. Elemental analyses (EA) were carried out on an Elementar UNICUBE microanalyzer. Fourier-transform infrared (FT-IR) spectra were collected on a Bruker Alpha II spectrometer in the 600–4000 cm^{-1} range. Cu K-edge X-ray absorption near-edge structure (XANES) spectra were measured at the TPS-44A beamline of the National Synchrotron Radiation Research Center (NSRRC, Taiwan).

Synthesis of Tetraamino-*p*-benzoquinone (TABQ)

TABQ was synthesized following a reported procedure.[1] In a 500 mL round-bottom flask, 35 g of tetraphthalimido-*p*-benzoquinone (TPBQ) was suspended in 125 mL hydrazine hydrate and heated at 65 °C for 2 h. After cooling to room temperature, the resulting mixture was filtered, and the residue was washed with DI water until the filtrate became colorless. A deep purple crystalline solid was obtained and dried under vacuum.

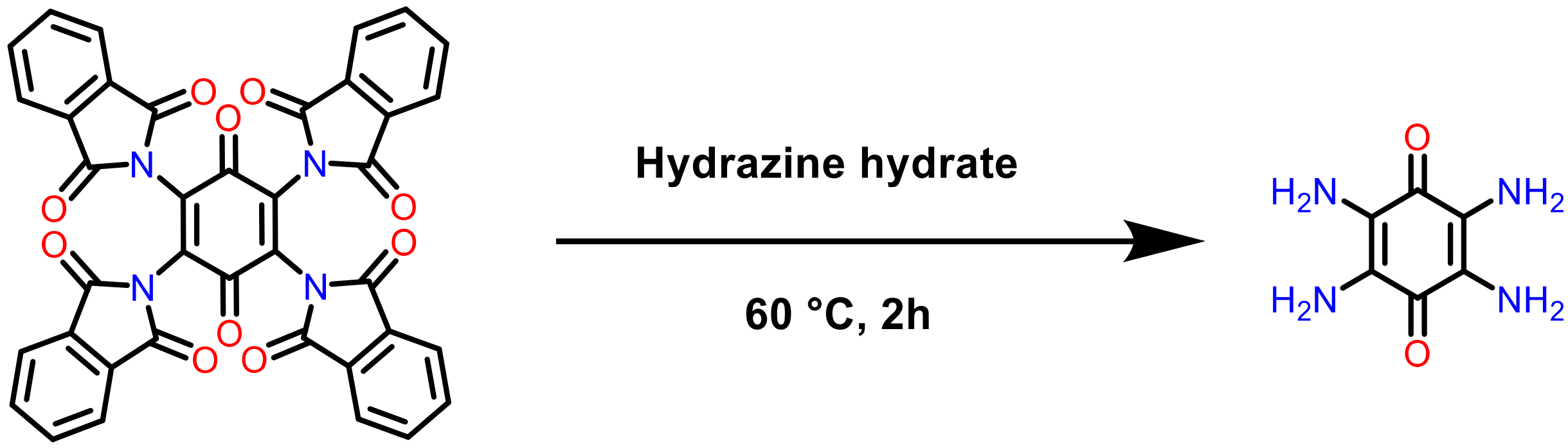

Synthesis of 2H,6H-benzo[1,2-d][4,5-d’]bistriazolequinone (H_2BBTQ)[1]

TABQ (1.2 g, 7.0 mmol) was dissolved in 24 mL of acetic acid while stirring. A solution of $NaNO_2$ (1.5 g, 23.0 mmol) in 28 mL of water was added dropwise over 10 min. The reaction mixture was stirred for 24 h at 25 °C, then filtered and washed three times with cold water. The crude product was recrystallized from boiling water, resulting in yellow crystals. The product was then dissolved in hot water (80 °C) and acidified with concentrated hydrochloric acid (HCl) to achieve a pH of 1. Yellow-green crystals of H_2BBTQ (0.6 g, 3.3 mmol, 47% yield) were collected by filtration and dried in a vacuum oven.

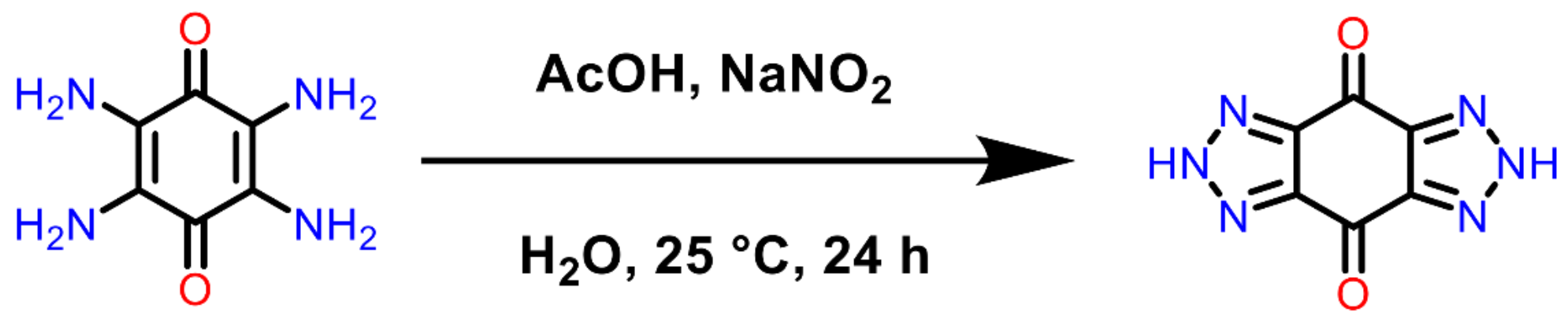

Supercritical CO_2 Drying

Supercritical CO_2 ($scCO_2$) drying was performed using the Tousimis Samdri PVT-3D critical-point dryer. The samples were soaked in absolute EtOH for 1 d, with the EtOH solution being replaced every 3 h. After removing the excess EtOH, the samples were transferred to the $scCO_2$ dryer. The chamber was filled with liquid CO_2 and maintained at 0 °C for 1 h. The samples were then subjected to a venting and bleeding process, repeated 3 times, with each cycle lasting 15 min. Once all the valves were closed, the temperature was raised to approximately 40 °C. After bleeding off the $scCO_2$ for 12 h, the samples were transferred to a glass vial under ambient atmosphere.

Fitting of CO_2 Adsorption Isotherm

The adsorption isotherm was fitted using the dual-site Langmuir–Freundlich (DSLF) model as previously published:[2]

$$q = q_A \frac{b_A P^{n_A}}{1 + b_A P^{n_A}} + q_B \frac{b_B P^{n_B}}{1 + b_B P^{n_B}}$$

In this context, *P* represents the absolute pressure measured in millimeters of mercury (mmHg). The variable *q* denotes the total adsorption capacity (cm^3 g^{-1}) at standard temperature and pressure (STP). The terms q_A and q_B refer to the adsorption capacities (cm^3 g^{-1} at STP) at sites A and B, respectively. The affinity coefficients (mmHg^{-1}) for these adsorption sites are represented by b_A for site A and b_B for site B. Finally, n_A and n_B indicate the degree of deviation from an ideal homogeneous surface at sites A and B.

Isosteric Heat of Adsorption (Q_{st})

The isosteric heat of adsorption (Q_{st}) was calculated from CO_2 isotherms collected at 273, 298, and 313 K using the Clausius–Clapeyron relation, as commonly applied in physisorption systems:[3]

$$Q_{st} = RT^2 \left(\frac{\partial \ln(P)}{\partial T}\right)_q$$

where R is the universal gas constant (8.314 J mol^{-1} K^{-1}), ln(P) is the natural logarithm of pressure (mmHg), and T is the temperature (K).

IAST Selectivity Calculation

Ideal adsorbed solution theory (IAST) selectivity for equimolar CO_2/N_2 mixtures was calculated using DSLF-fitted single-component gas adsorption isotherms.[2] The selectivity (S) is defined as:[4]

$$S = \frac{(x_1/y_1)}{(x_2/y_2)}$$

Where x_i and y_i are the mole fractions of component i in the adsorbed and gas phases, respectively, under equilibrium conditions.

Evaluation of Brunauer–Emmett–Teller (BET) Surface Area

The apparent BET surface area was calculated from the linear region of the BET plot satisfying the Rouquerol criteria[5]:

$$\frac{P/P_0}{V(1 - P/P_0)} = \frac{1}{V_m C} + \frac{C - 1}{V_m C}\left(\frac{P}{P_0}\right)$$

V: Volume of gas adsorbed at standard temperature and pressure ($cm^3\ g^{-1}$).

Vm: Monolayer capacity of adsorbed gas ($cm^3\ g^{-1}$).

C: BET constant.

The cross-sectional area of the CO_2 probe molecule was taken as 0.170 nm^2.

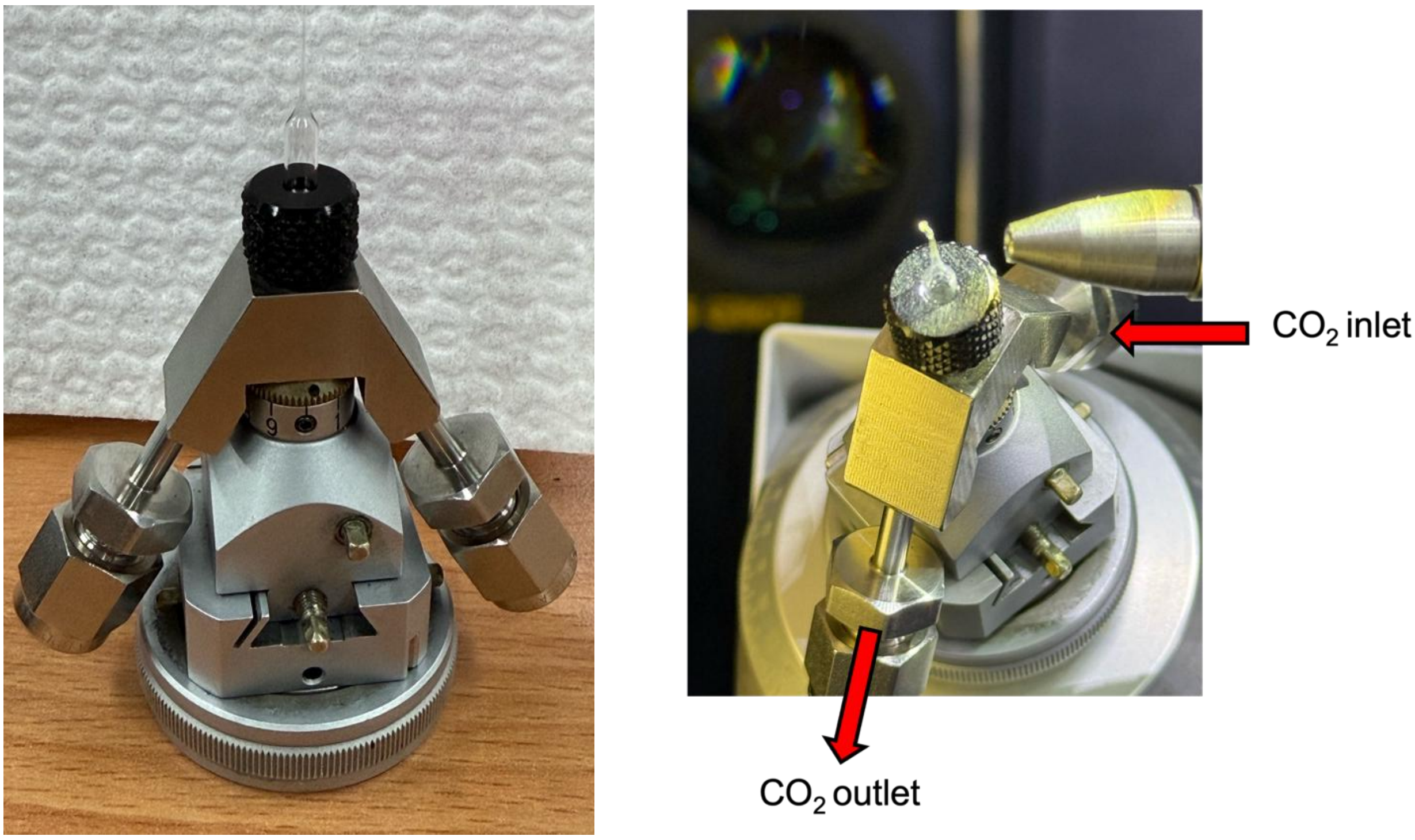

Figure S1. Customized goniometer head for single-crystal X-ray diffraction analysis upon CO_2 adsorption. The gas loading holder was designed in collaboration with GINGEN TECHNOLOGY CO., LTD.

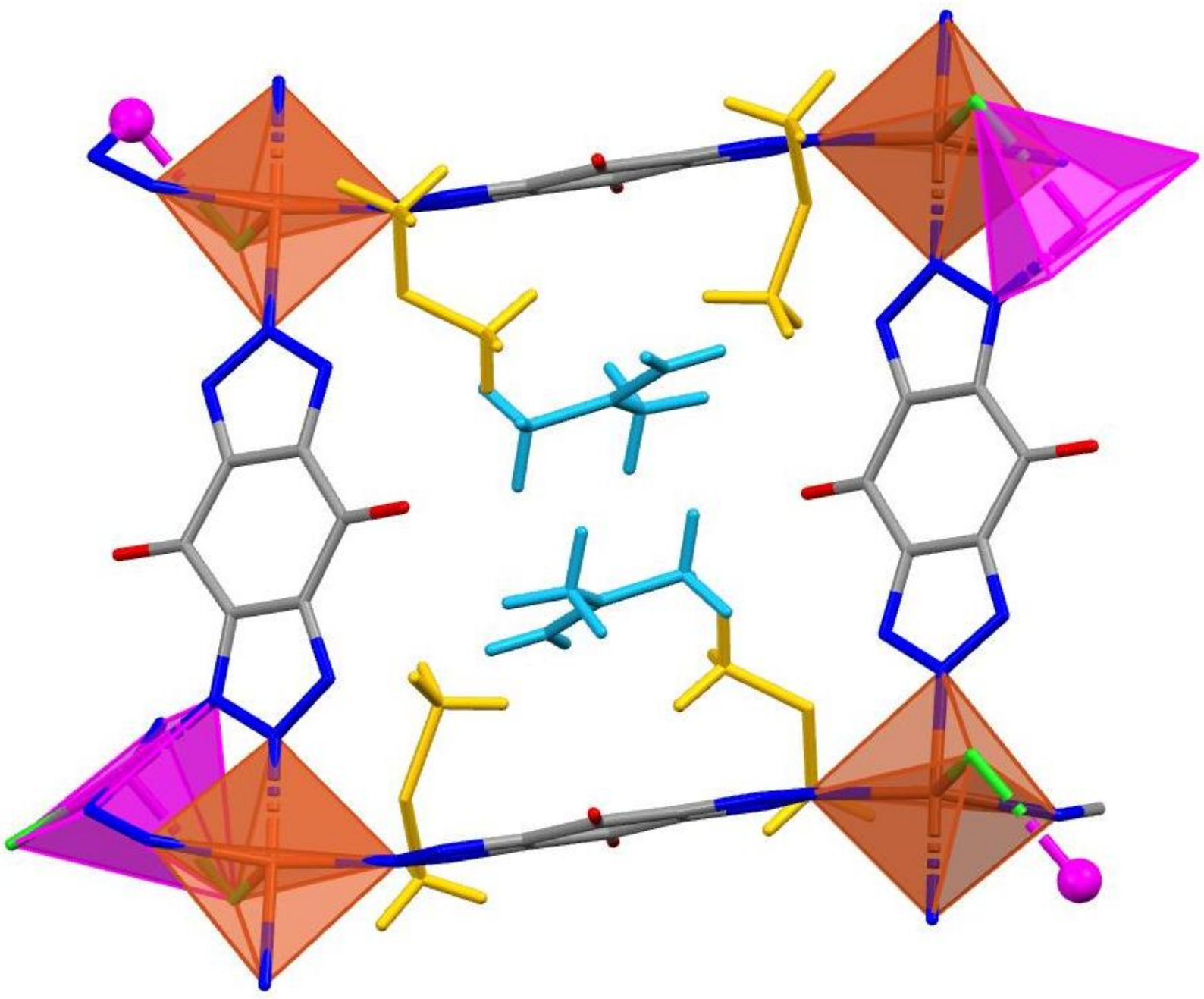

Figure S2. Crystal structure of NCKU-56 with $[NH_2(CH_3)_2]^+$ cations (yellow) and trapped DMF molecules (cyan). Element colors: C—gray, O—red, N—blue, Cl— light green, and Cu—brown and magenta.

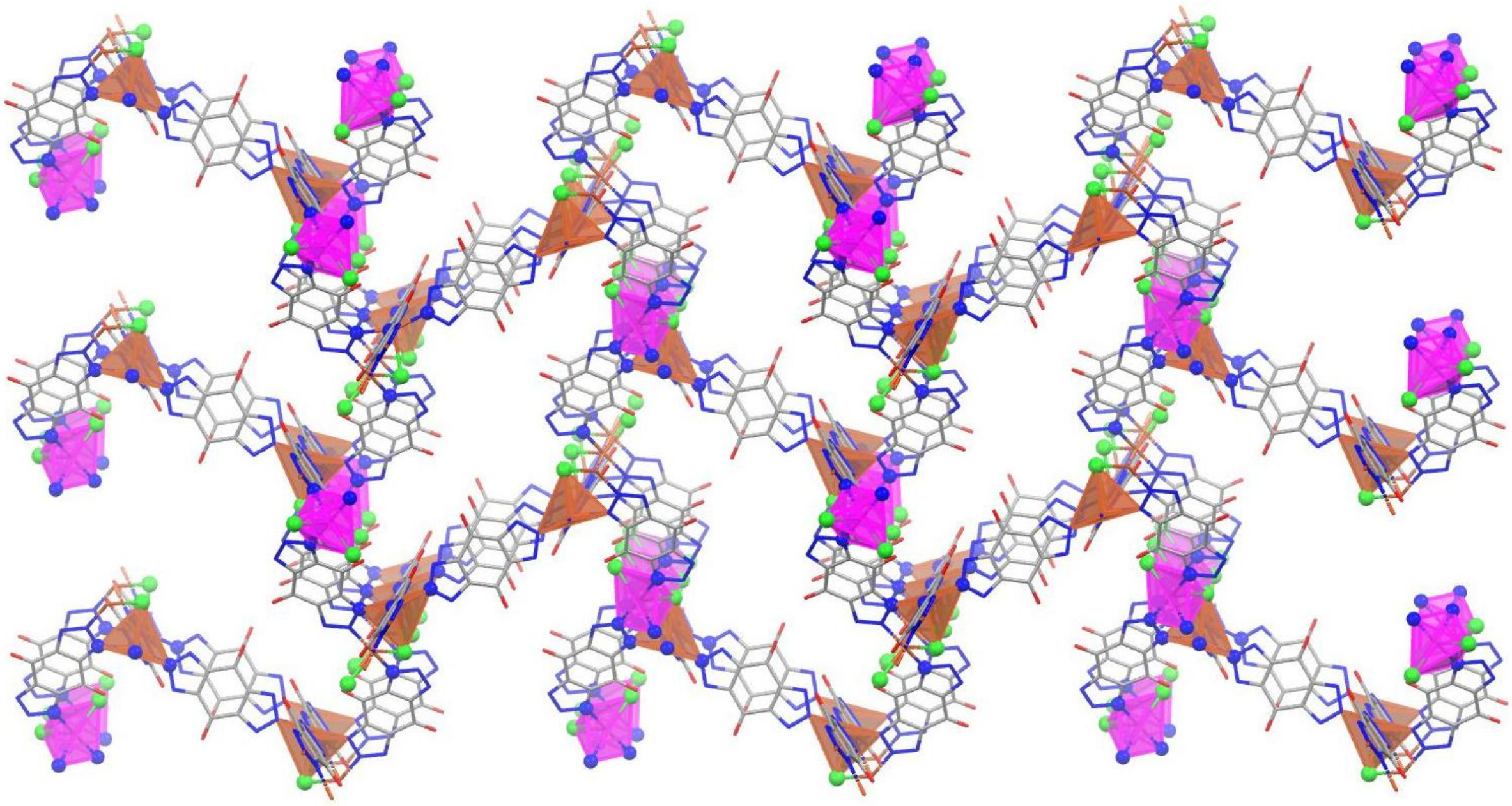

Figure S3. Crystal Structure of NCKU-56 along the *a*-axis. Solvent molecules and $[NH_2(CH_3)_2]^+$ cations are omitted for clarity. Element colors: C—gray, O—red, N—blue, Cl— light green, and Cu—brown and magenta.

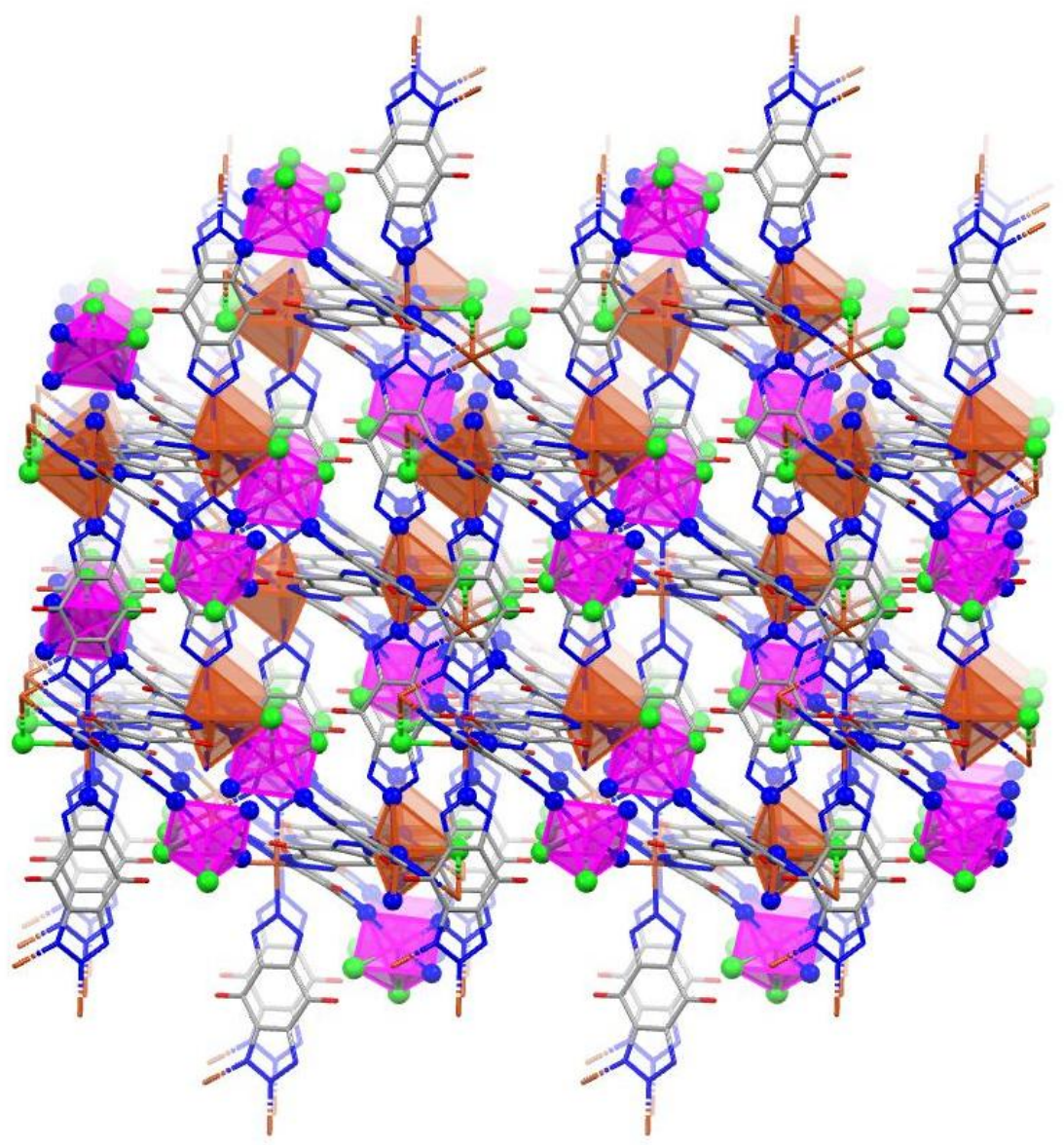

Figure S4. Crystal Structure of NCKU-56 along the *b*-axis. Solvent molecules and $[NH_2(CH_3)_2]^+$ cations are omitted for clarity. Element colors: C—gray, O—red, N—blue, Cl— light green, and Cu—brown and magenta.

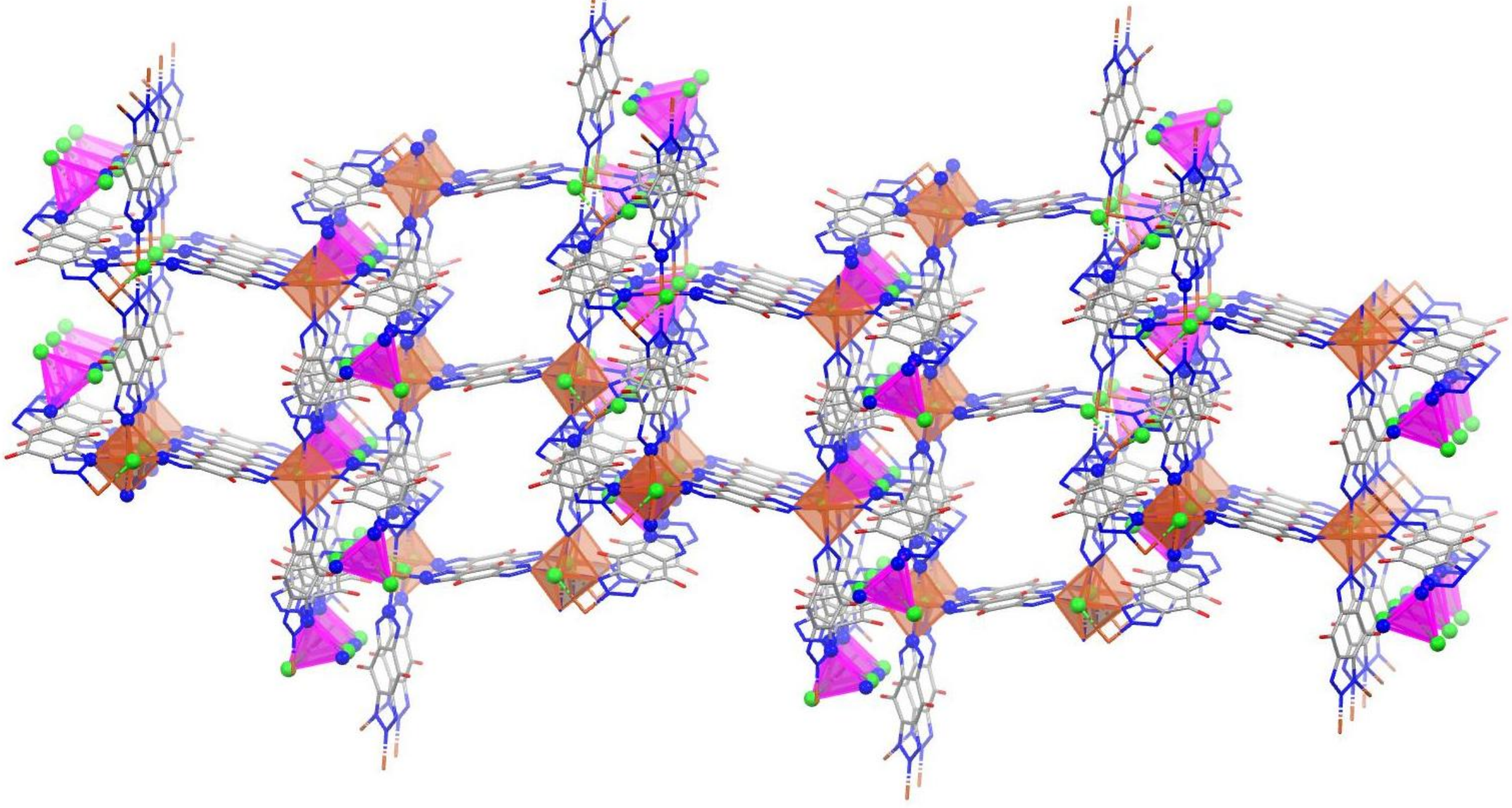

Figure S5. Crystal Structure of NCKU-56 along the *c*-axis. Solvent molecules and $[NH_2(CH_3)_2]^+$ cations are omitted for clarity. Element colors: C—gray, O—red, N—blue, Cl— light green, and Cu—brown and magenta.

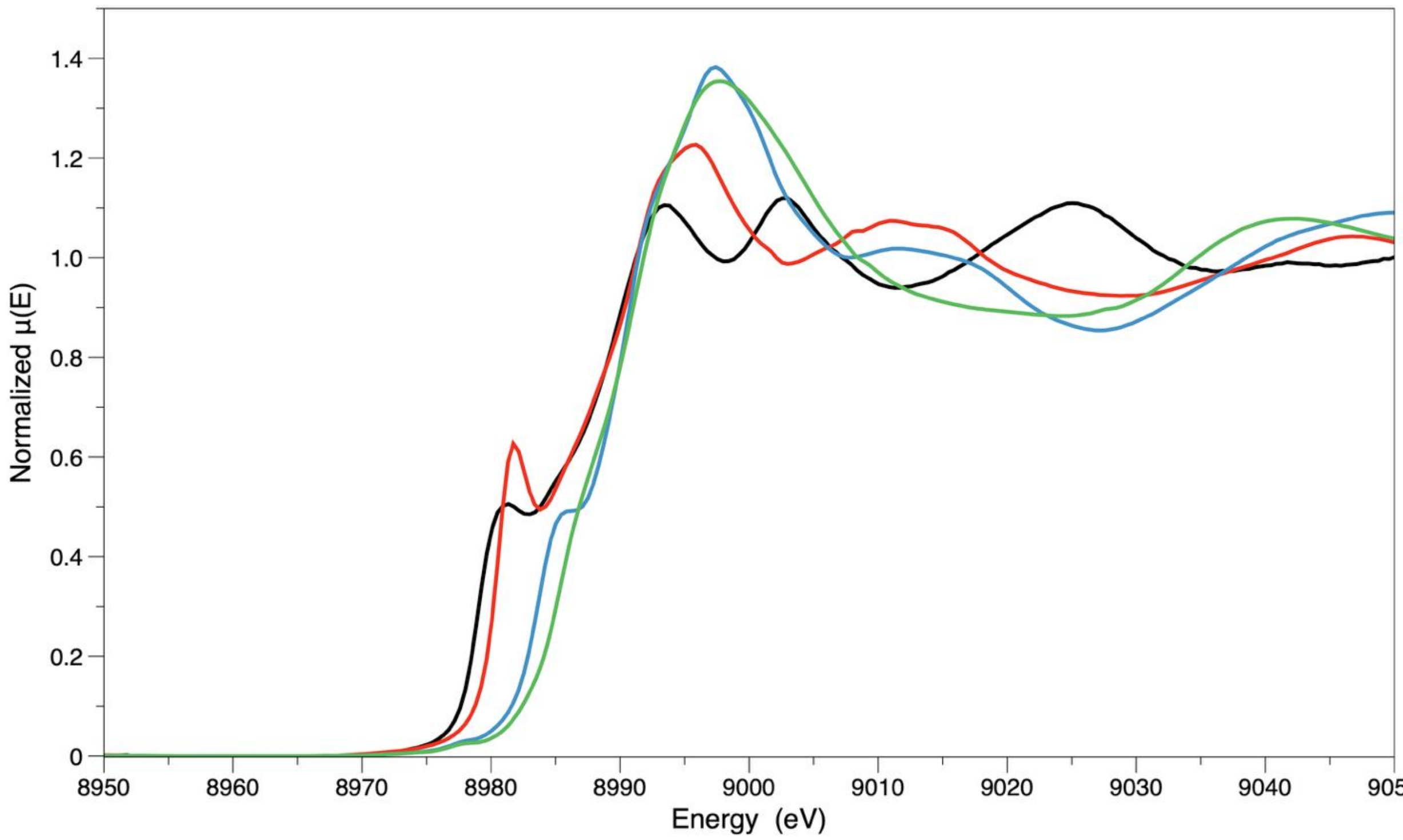

Figure S6. Cu K-edge XANES patterns of NCKU-56 (green), Cu foil (black), Cu_2O (red), and CuO (blue).

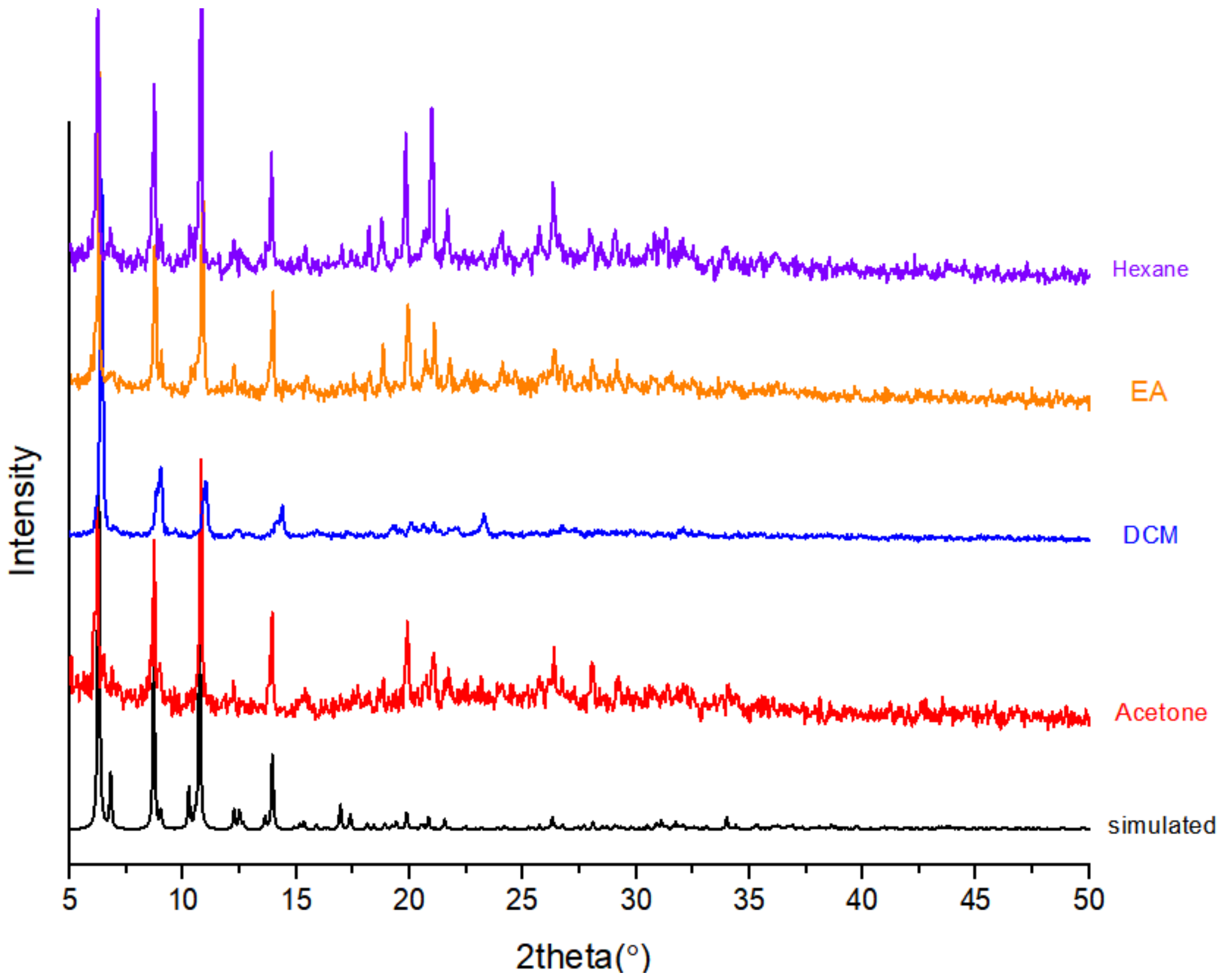

Figure S7. PXRD patterns of NCKU-56 soaking in different solvents for 1 d.

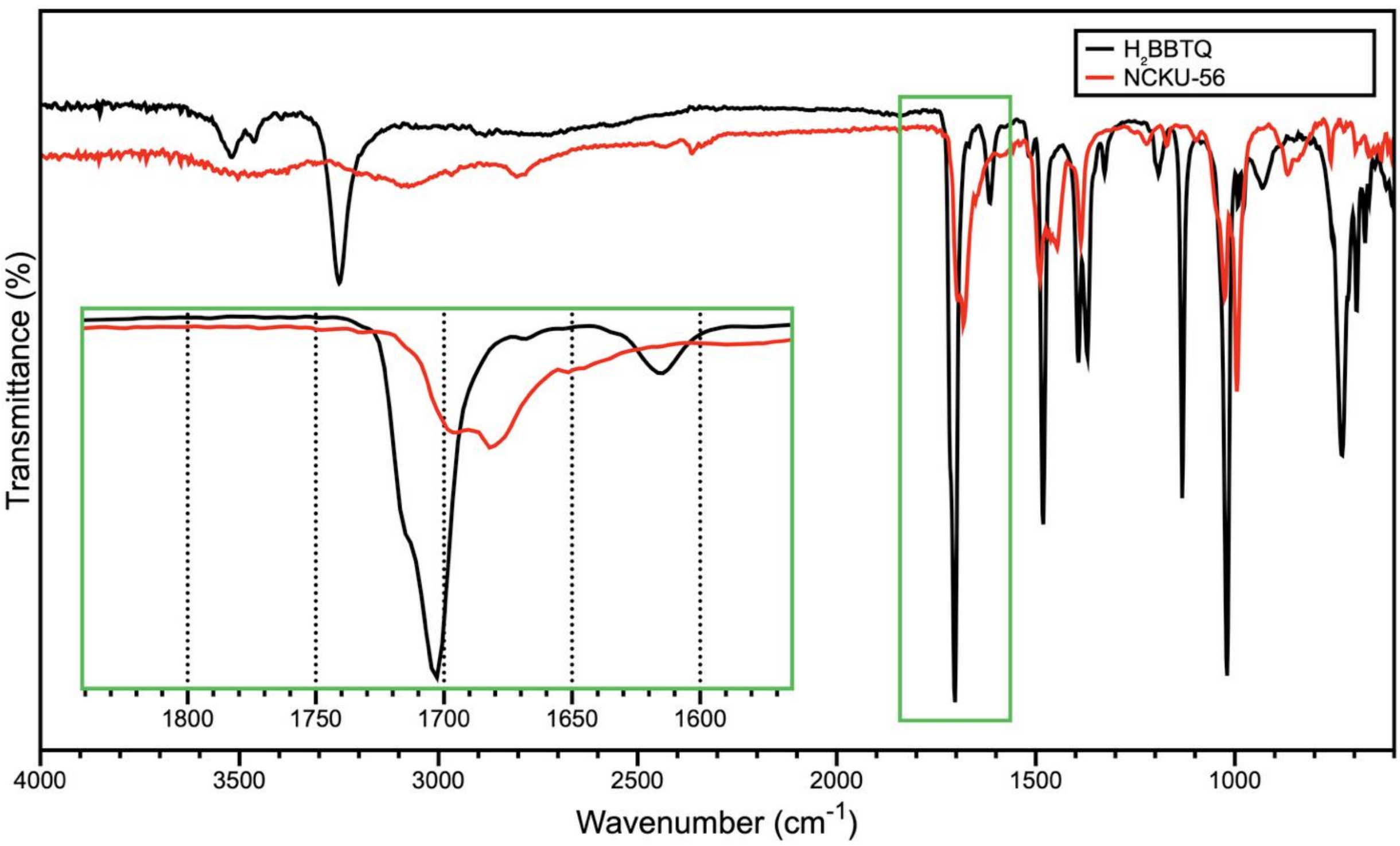

Figure S8. FT-IR spectra of H_2BBTQ and NCKU-56.

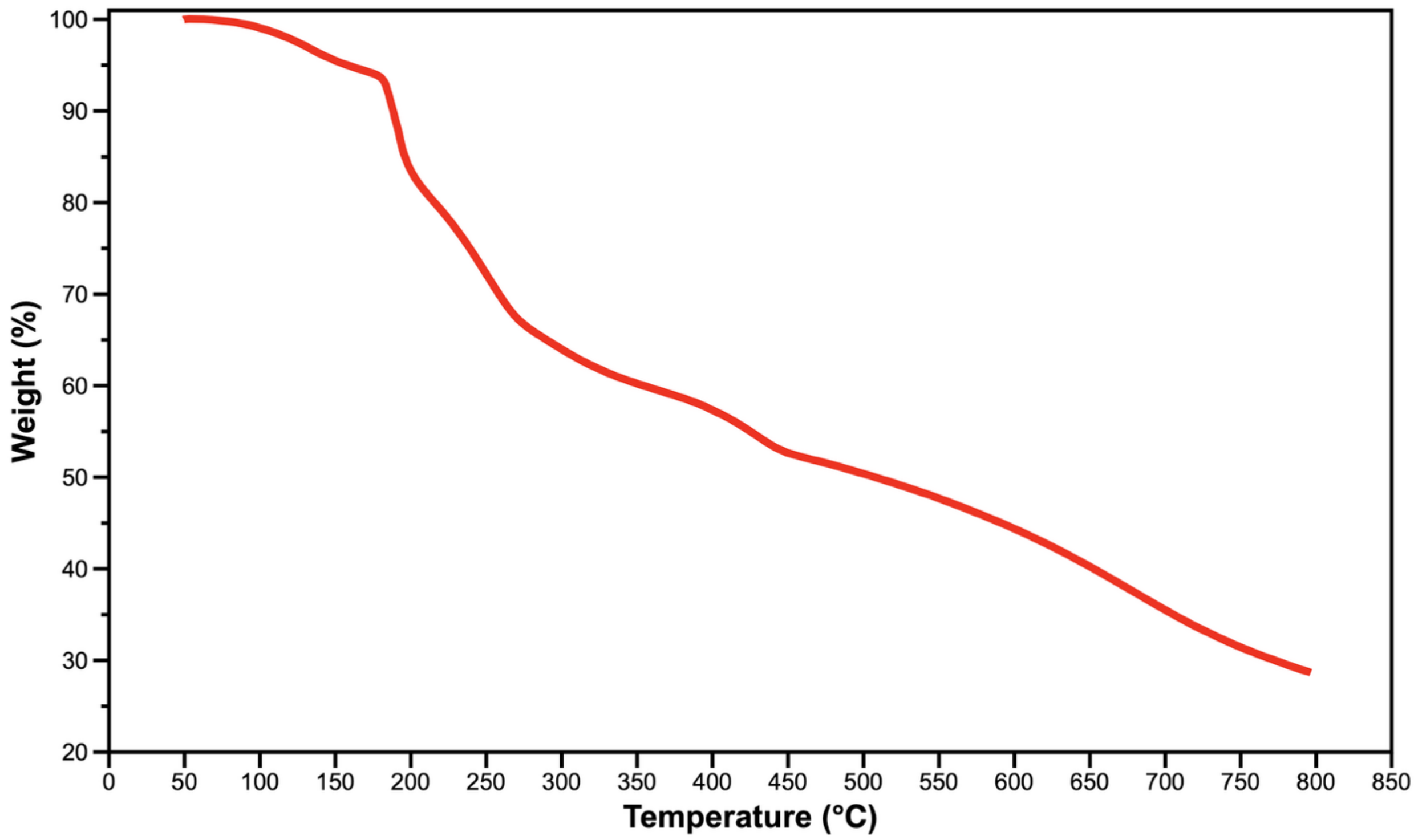

Figure S9. TGA curve of as-synthesized NCKU-56.

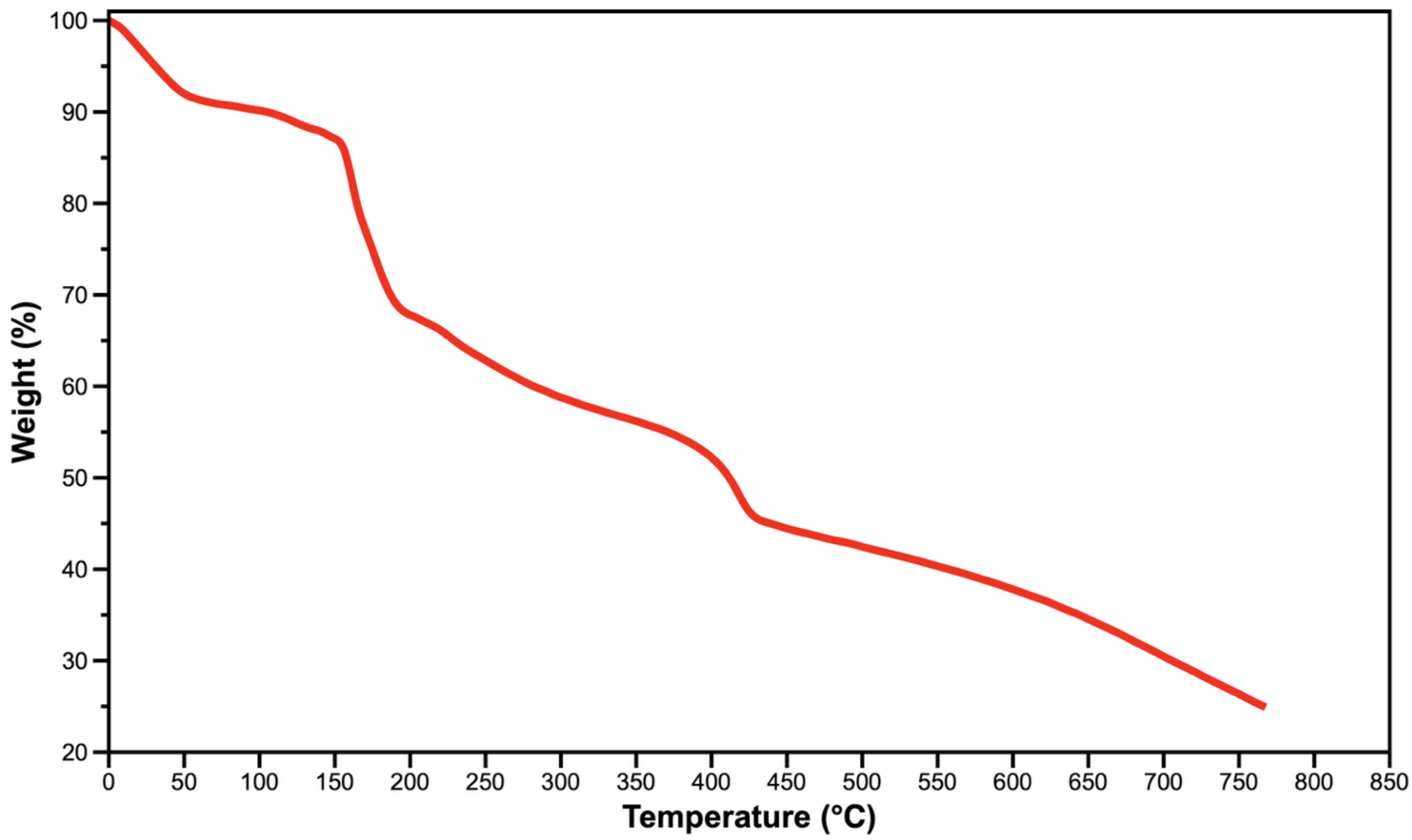

Figure S10. TGA curve of $scCO_2$-activated NCKU-56.

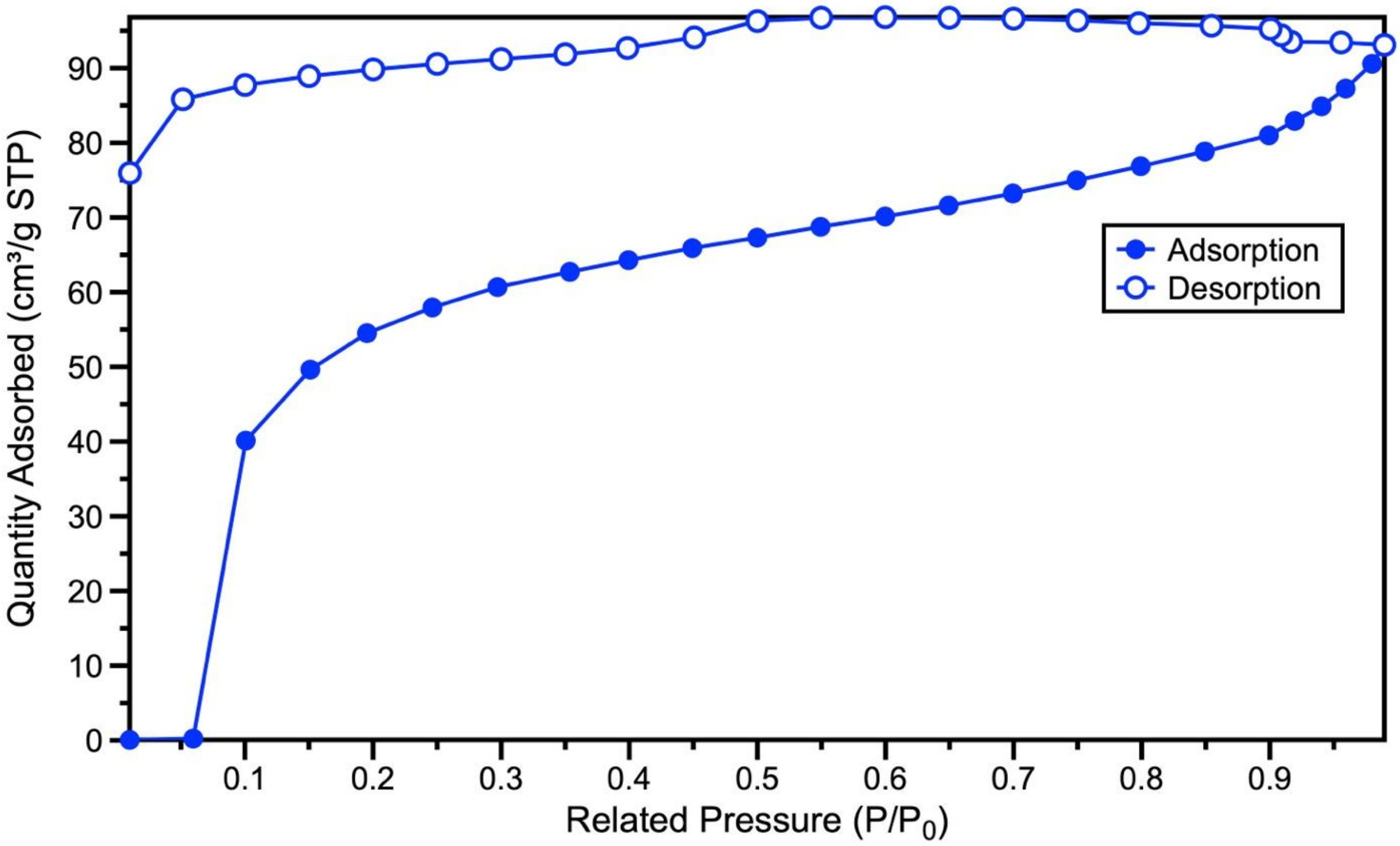

Figure S11. N_2 adsorption–desorption isotherms of NCKU-56 at 77 K.

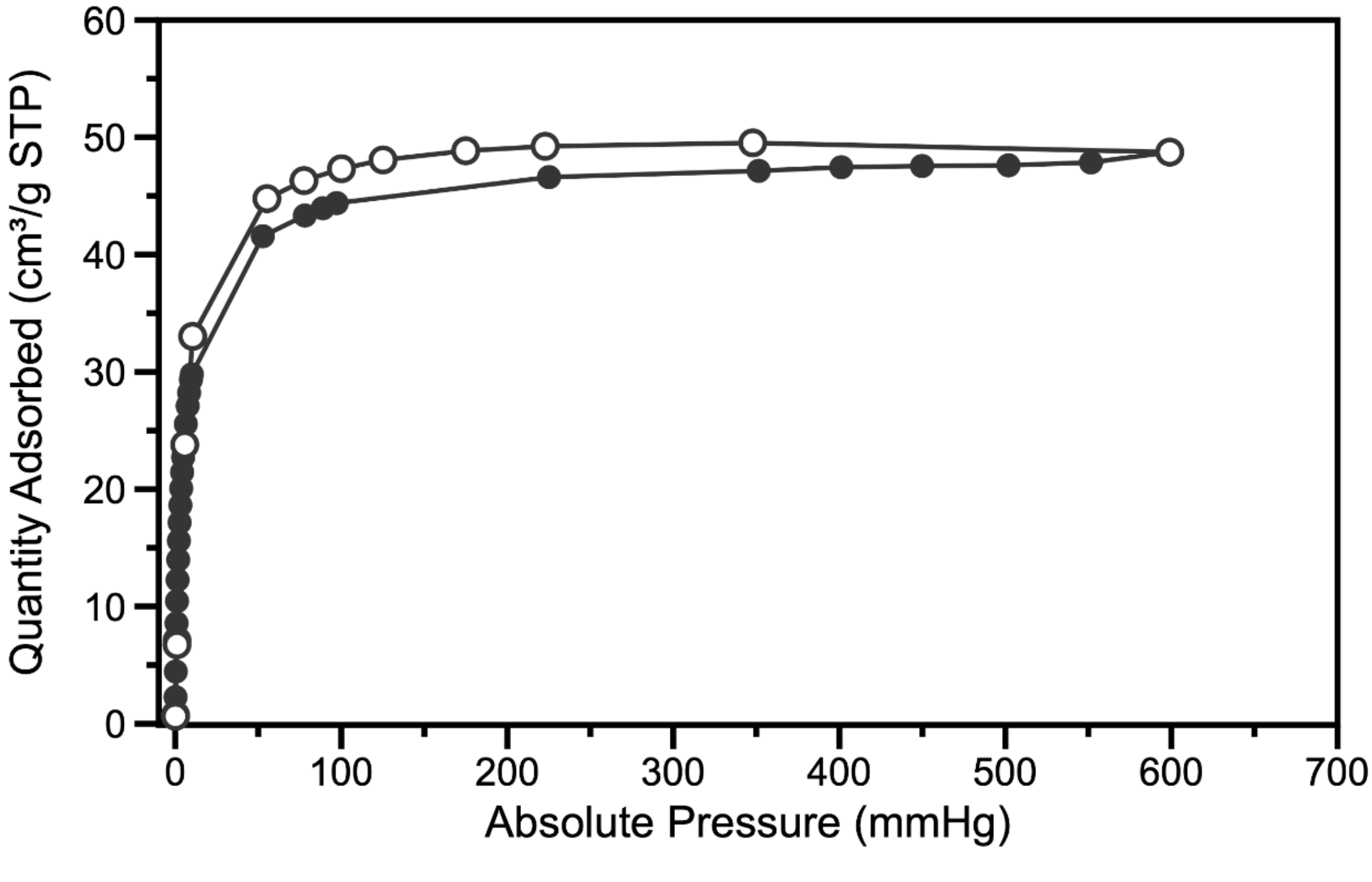

Figure S12. CO_2 adsorption–desorption isotherms of NCKU-56 at 195 K.

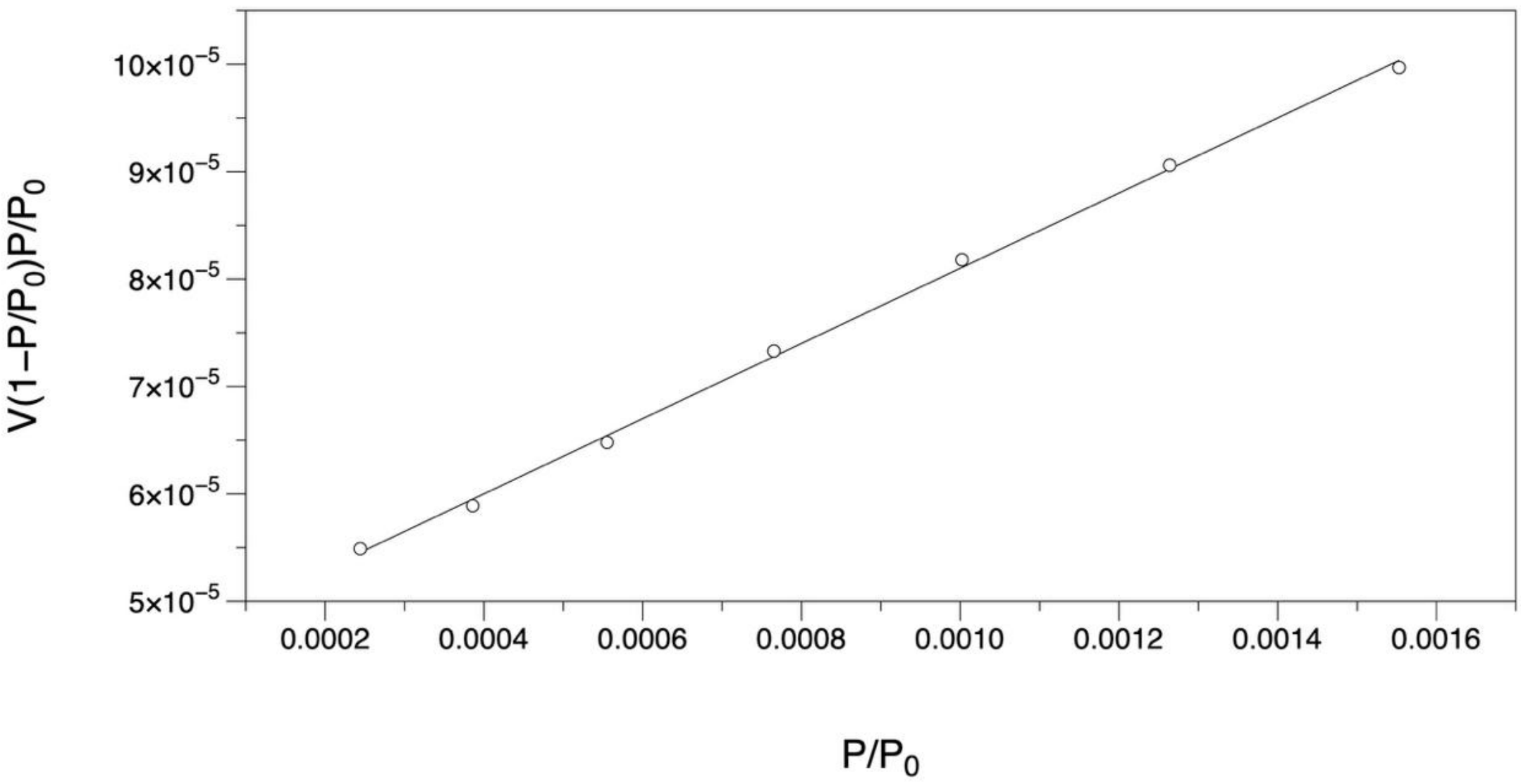

Figure S13. The linear BET plot of the 195 K CO_2 adsorption isotherm in the selected P/P_0 range.

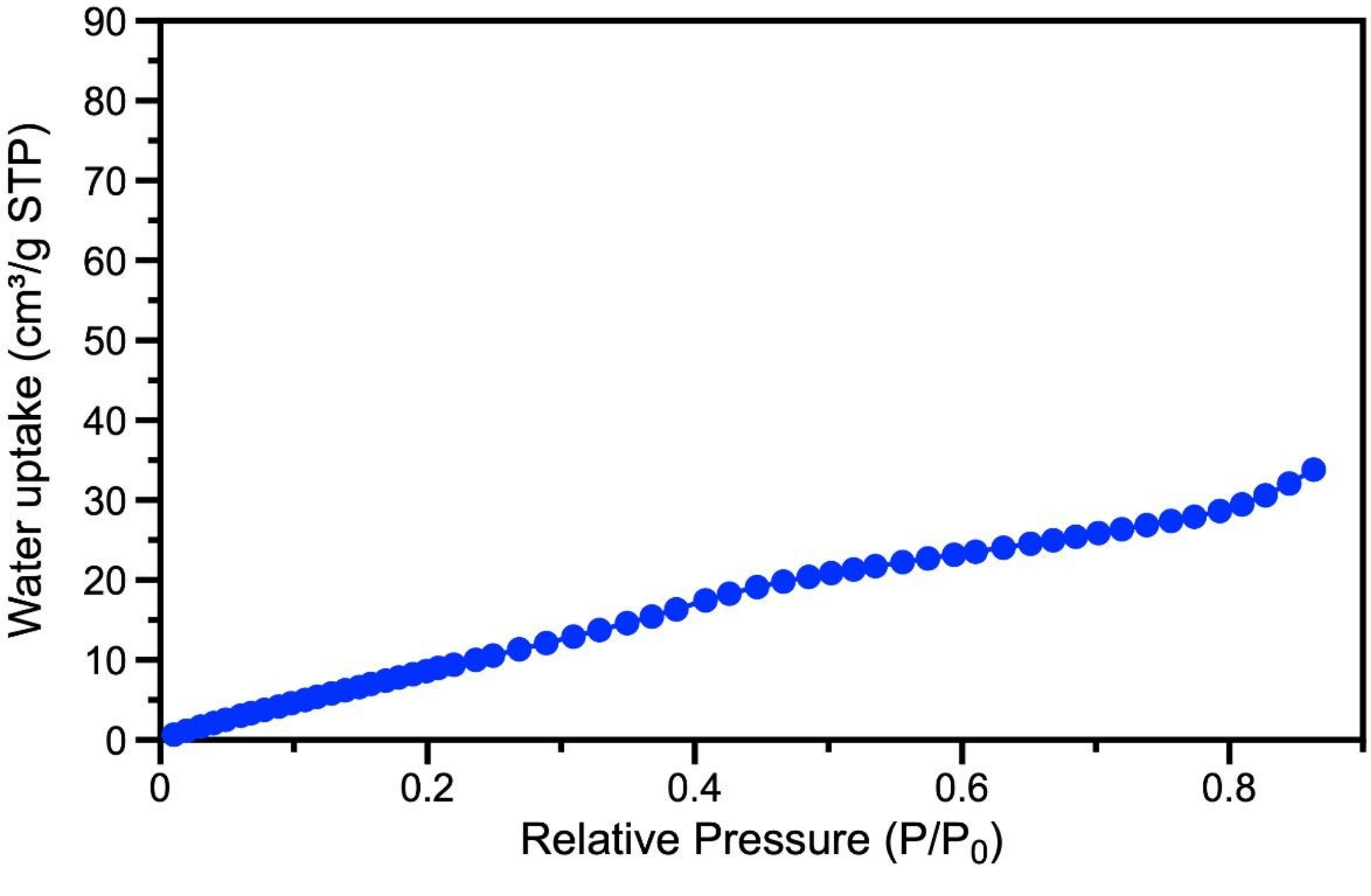

Figure S14. Water vapor adsorption isotherm of NCKU-56 at 298 K.

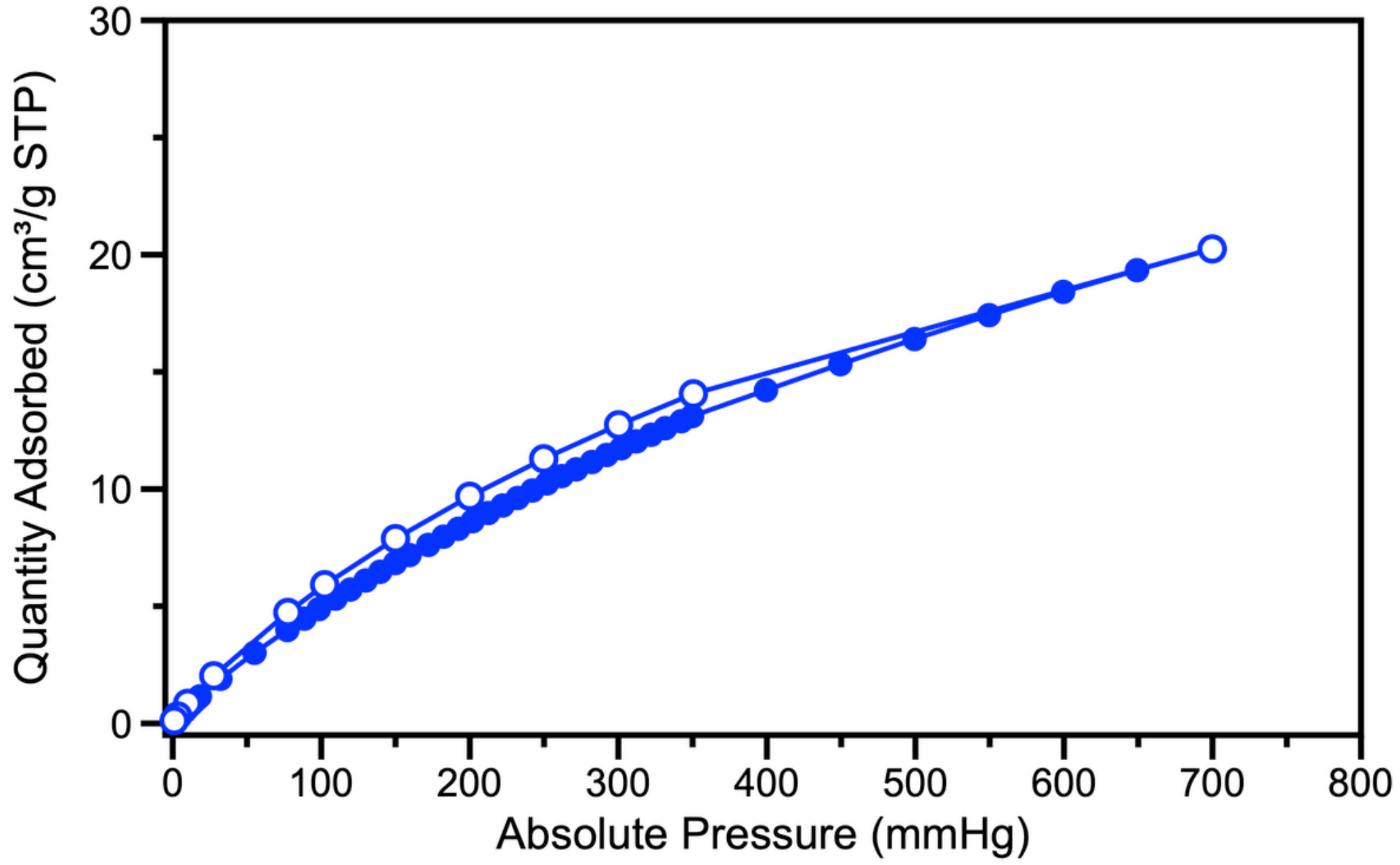

Figure S15. CO_2 sorption isotherm of NCKU-56 at 298 K after moisture exposure for 1 d.

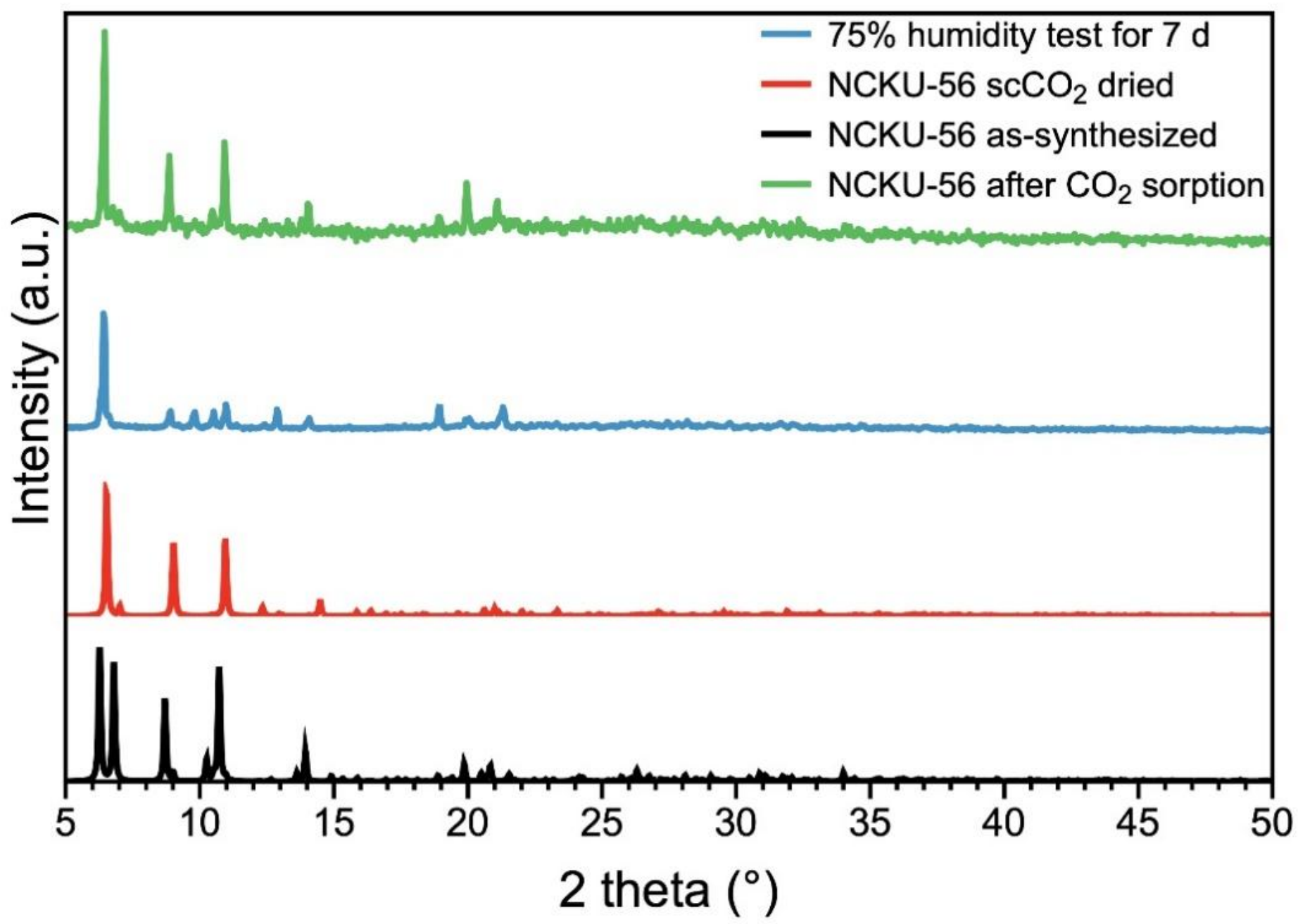

Figure S16. Simulated PXRD patterns of as-synthesized and $scCO_2$-dried NCKU-56, and experimental PXRD patterns of NCKU-56 after CO_2 adsorption and after exposure to a humid environment (RH = 75%) for 7 d.

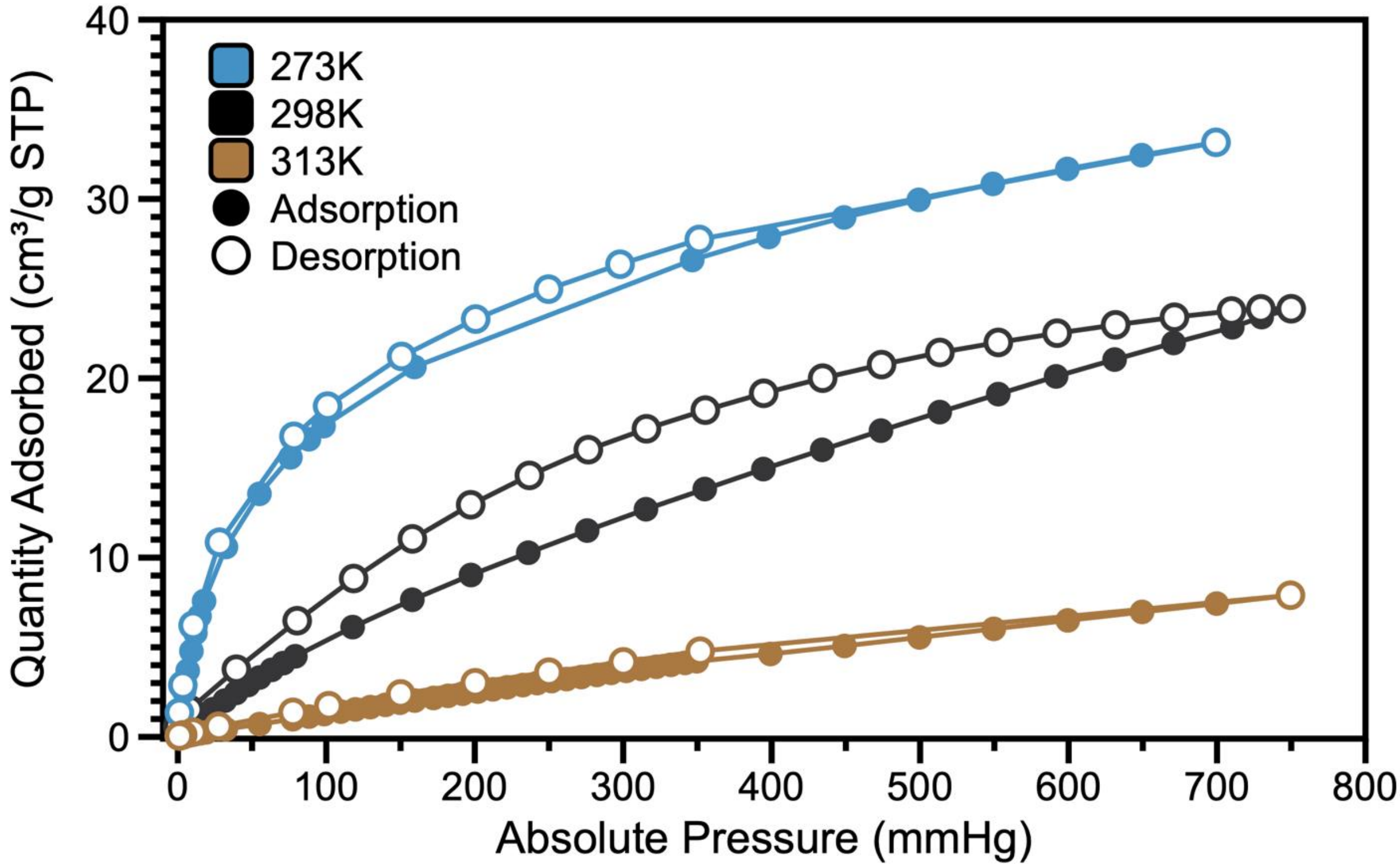

Figure S17. CO_2 sorption isotherms of NCKU-56 at 273 K, 298 K, and 313 K.

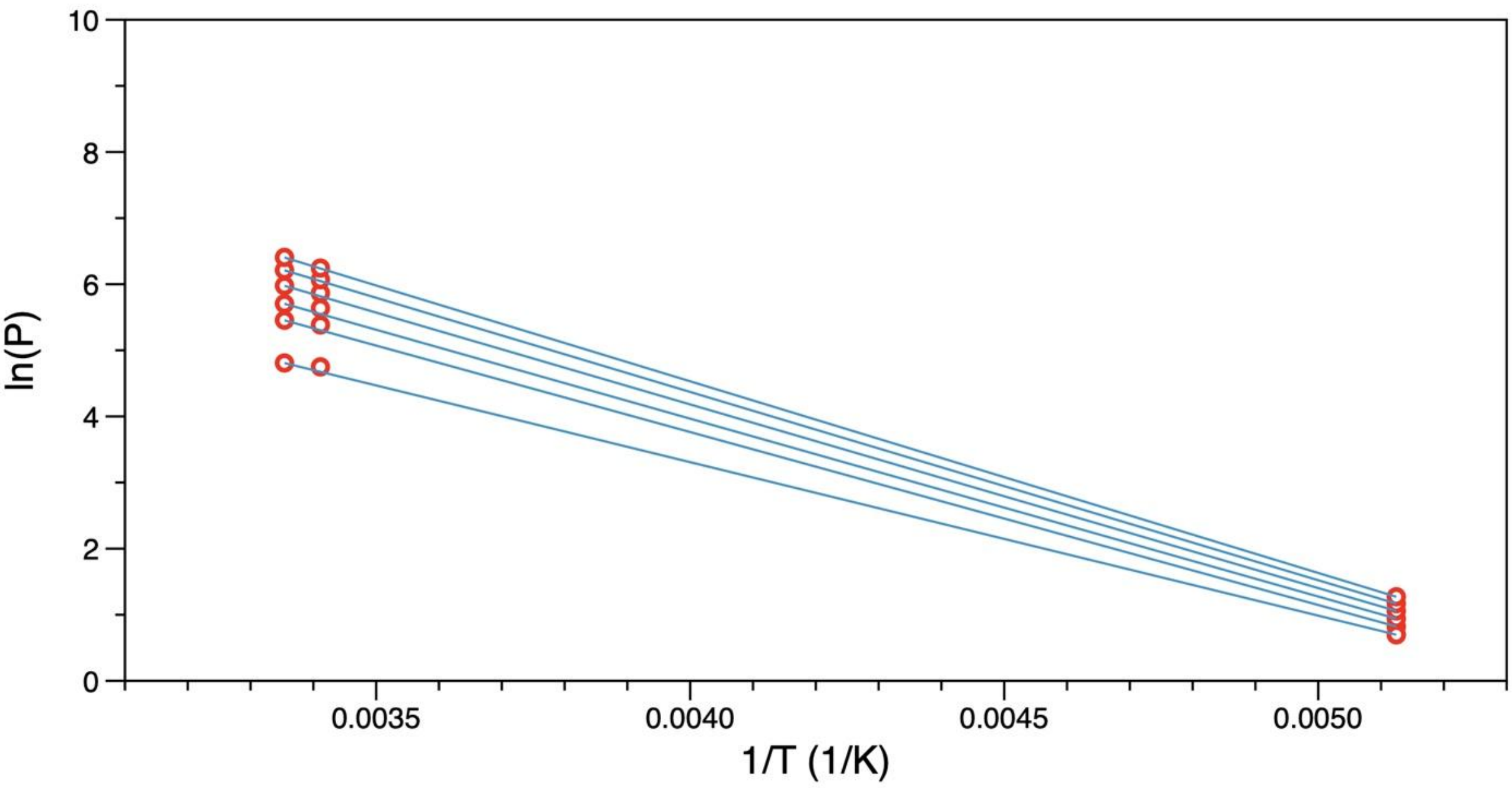

Figure S18. CO_2 sorption isostere plot (ln(P) against 1/T) of NCKU-56 at 273, 298, and 313 K for the calculation of Qst.

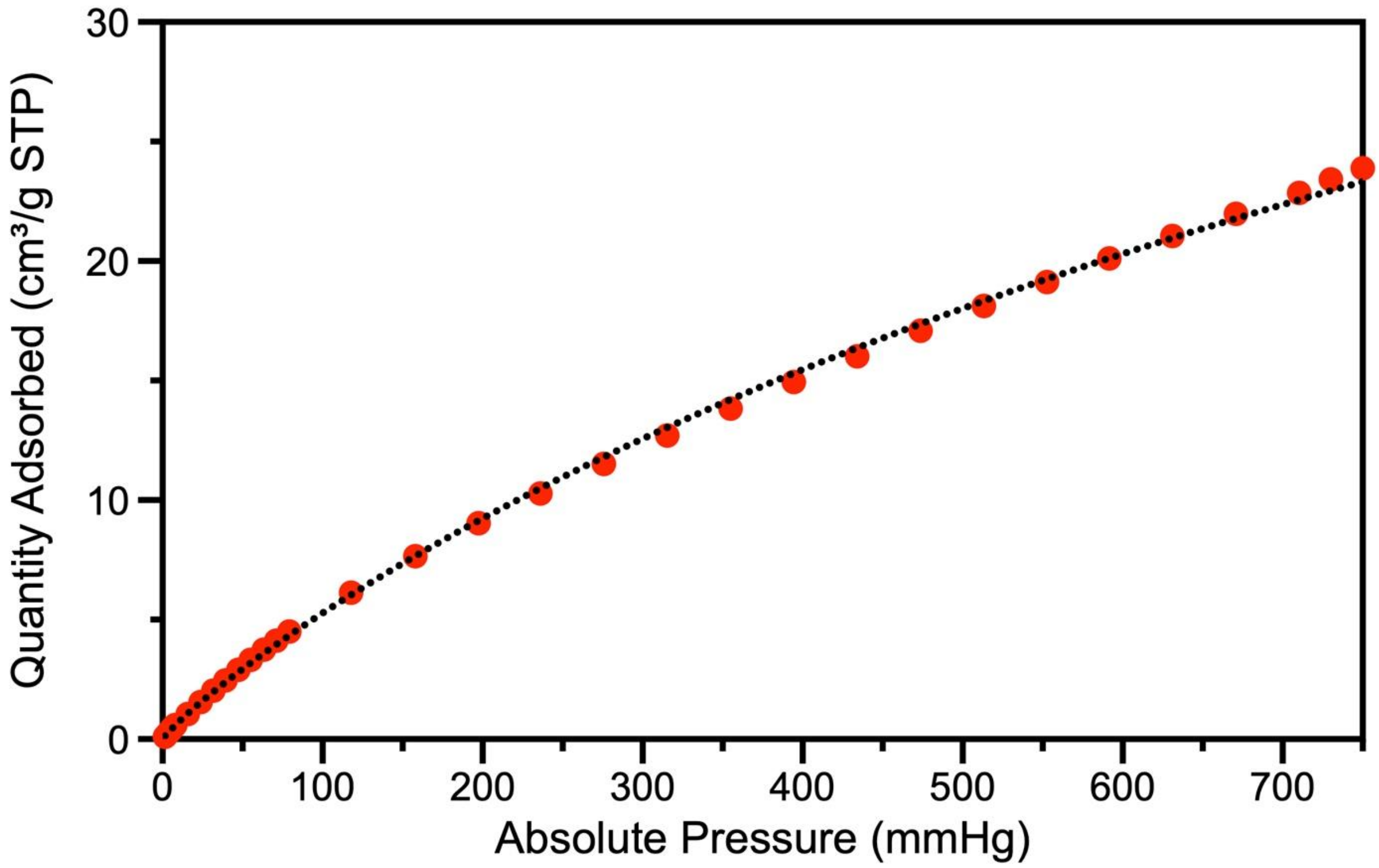

Figure S19. Experimental (red point) and DSLF-fitted (dashed line) CO_2 adsorption isotherms of NCKU-56 at 298 K.

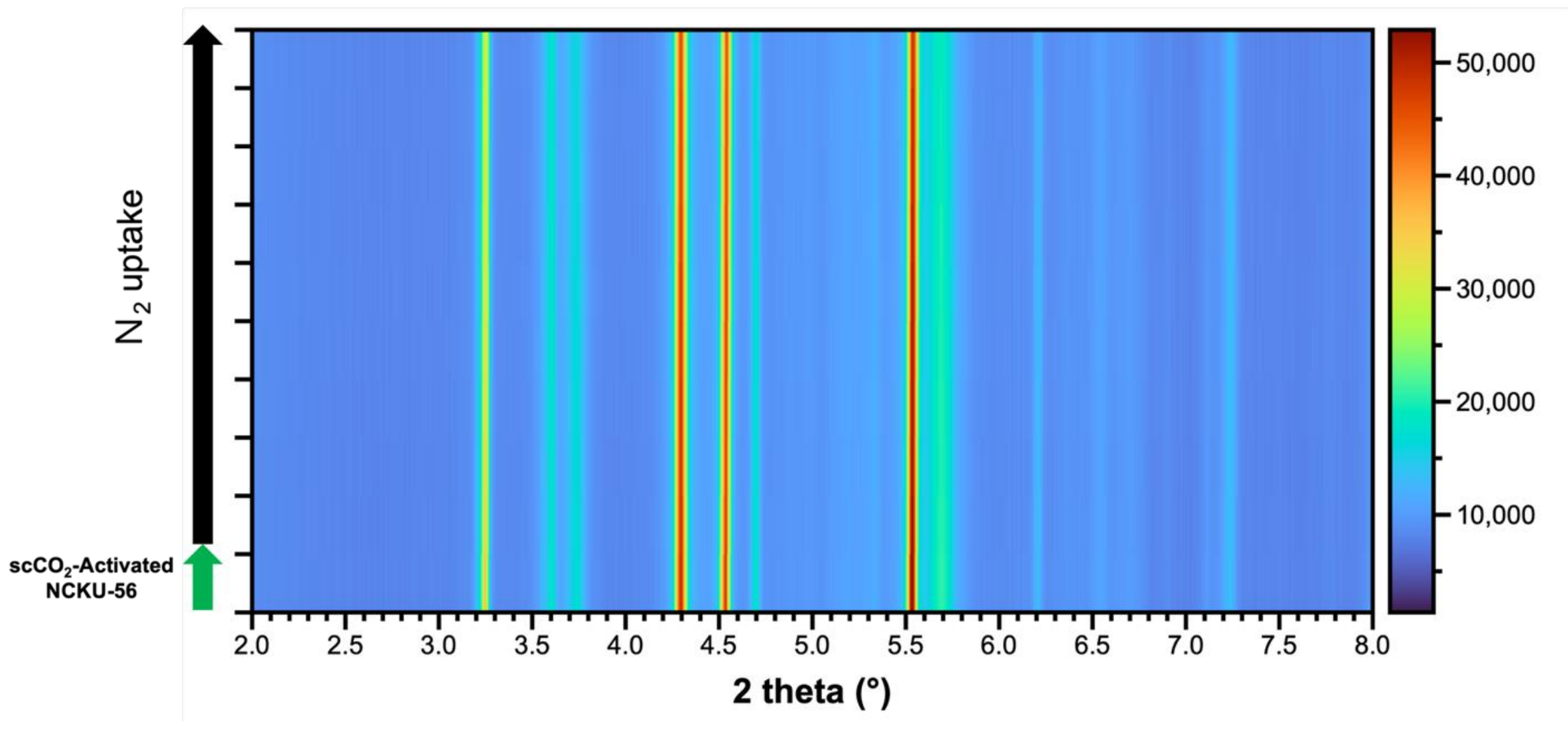

Figure S20. *In situ* synchrotron PXRD analysis of $scCO_2$-activated NCKU-56 during an N_2 flow with data collected every 3 min.

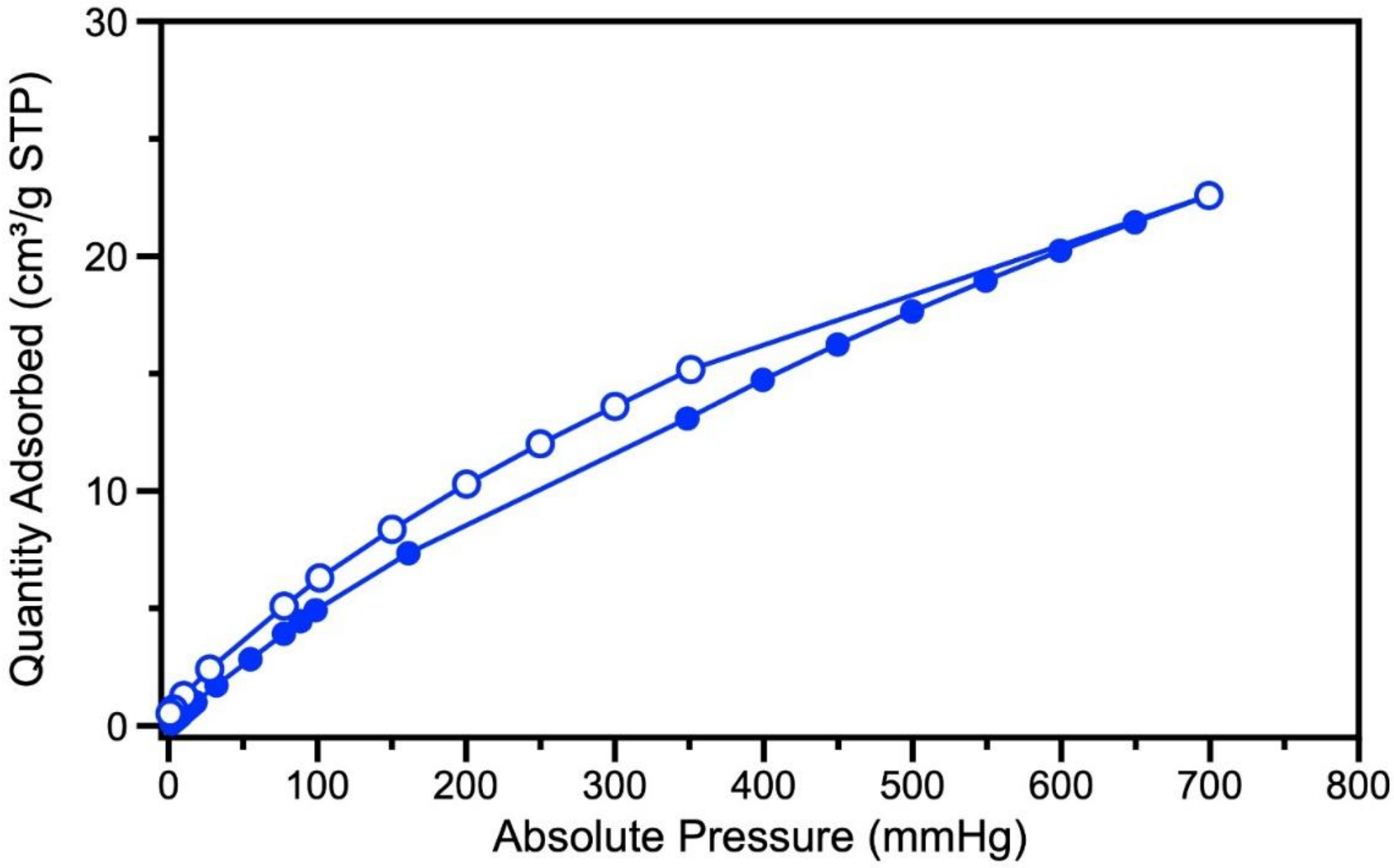

Figure S21. Full CO_2 adsorption (closed circles) and desorption (open circles) isotherms of NCKU-56 measured at 298 K after 5 adsorption-desorption tests.

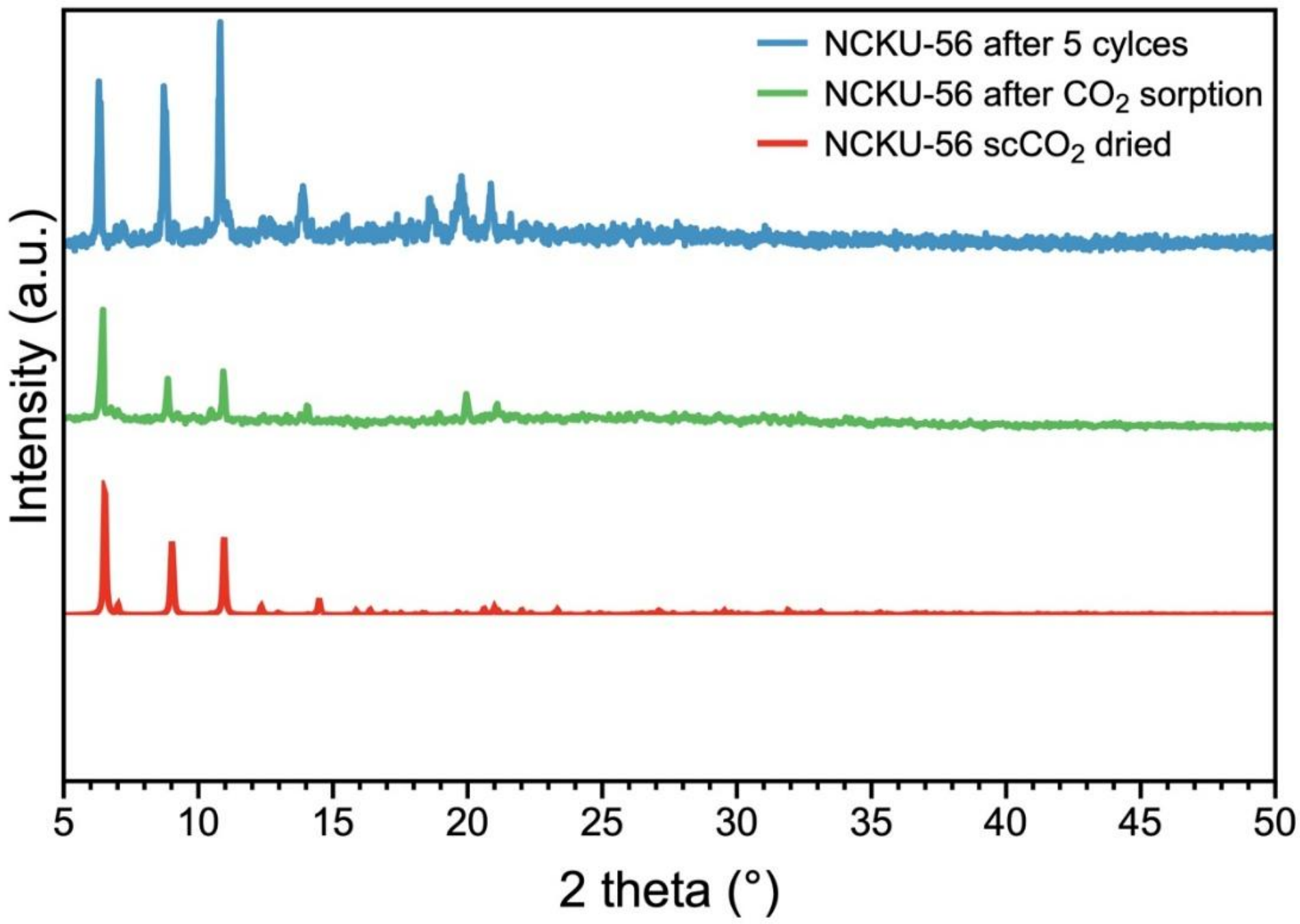

Figure S22. Simulated PXRD patterns of $scCO_2$-dried NCKU-56, and experimental PXRD patterns of NCKU-56 after CO_2 adsorption and after 5 cycles of CO_2 adsorption.

Table S1. Crystallographic Data of As-synthesized NCKU-56

Empirical formula	$C_{48}H_{50}Cl_4Cu_4N_{38}O_{12}$	
Formula weight	1747	
Temperature	200(2) K	
Wavelength	0.71073 Å	
Crystal system	Monoclinic	
Space group	$P2_1/c$	
Unit cell dimensions	a = 10.46 Å	α = 90°
	b = 28.17 Å	β = 91.5°
	c = 14.65 Å	γ = 90°
Volume	4316.7 $Å^3$	
Z	2	
Density (calculated)	1.344 mg/cm^3	
Crystal size	0.190×0.070 ×0.010 (mm)	
Reflections collected	9930	
Independent reflections	7646 [R_{int} = 0.0438]	
Data/restraints/parameters	7646 / 4 / 483	
Goodness-of-fit on F^2	0.989	
Final R indexes [$I \geq 2\sigma (I)$]	R_1 = 0.0485, wR_2 = 0.1205	
R indexes (all data)	R_1 = 0.0654, wR_2 = 0.1294	

Table S2. Crystallographic Data of $scCO_2$-Activated NCKU-56

Empirical formula	$C_{21}H_{21}Cl_2Cu_2N_{18}O_5$	
Formula weight	803.54	
Temperature	100 K	
Wavelength	0.71073 Å	
Crystal system	Monoclinic	
Space group	$P2_1/c$	
Unit cell dimensions	a = 10.45 Å	α = 90°
	b = 27.05 Å	β = 90.46°
	c = 14.22 Å	γ = 90°
Volume	4020.99 $Å^3$	
Z	4	
Density (calculated)	1.327 Mg/cm^3	
Crystal size	0.240×0.109×0.038 (mm)	
Reflections collected	9893	
Independent reflections	10000 [R_{int} = 0.0505]	
Data/restraints/parameters	10000 / 0 / 438	
Goodness-of-fit on F^2	1.074	
Final R indexes [$I \geq 2\sigma(I)$]	R_1 = 0.0533, wR_2 = 0.1274	
R indexes (all data)	R_1 = 0.0646, wR_2 = 0.1226	
Largest diff. peak/hole	1.294 / -0.781 e $Å^{-3}$	

Table S3. Crystallographic Data of CO_2-loaded NCKU-56 upon CO_2 Uptake

Empirical formula	$C_{96}H_{98}Cl_8Cu_8N_{74}O_{22}$	
Formula weight	3449	
Temperature	102 K	
Wavelength	0.71073 Å	
Crystal system	Monoclinic	
Space group	$P2_1/c$	
Unit cell dimensions	a = 10.45 Å	α = 90°
	b = 23.28 Å	β = 92.26°
	c = 14.16 Å	γ = 90°
Volume	3443.03 $Å^3$	
Z	1	
Density (calculated)	1.664 Mg/cm^3	
Crystal size	0.137×0.057×0.031 (mm)	
Reflections collected	9820	
Independent reflections	8528 [R_{int} = 0.0505]	
Data/restraints/parameters	8528 / 1.111 / 529	
Goodness-of-fit on F^2	1.115	
Final R indexes [$I \geq 2\sigma(I)$]	R_1 = 0.1100, wR_2 = 0.2848	
R indexes (all data)	R_1 = 0.1310, wR_2 = 0.2981	

Table S4. Parameters Derived from the CO_2 Adsorption Isotherms of NCKU-56 at 195 K[6]

Parameter	Value
Selected P/P_0 range	2.44×10^{-4} to 1.55×10^{-3}
Slope (s)	0.03957
Intercept (i)	2.69×10^{-5}
Monolayer capacity (V_m)	25.26 $cm^3\ g^{-1}$
BET constant (C)	1469.7
Correlation coefficient (R^2)	0.9998
BET Surface Area (S_{BET})	192.5 $m^2\ g^{-1}$

Table S5. Comparison of CO_2 Adsorption Capacity and CO_2/N_2 (15:85) IAST Selectivity for NCKU-56 and Reference MOF Materials

	Material	CO_2 Uptake at 298 K, 1 bar ($cm^3\ g^{-1}$)	CO_2/N_2 (15:85) IAST Selectivity	Ref
**1**	**NCKU-56**	**24.8**	**315**	**This work**
**2**	IITKGP-11	60.5	52.27	7
**3**	$\{[Co(L)_{0.5}(OBA)]\cdot xG\}_n$	68.0	31.7	8
**4**	IITKGP-12	66.8	75	9
**5**	IITKGP-8	26.5	43.7	10
**6**	IITKGP-21	42.2	16.4	11
**7**	ZJNU-40	108.0	22.9	12
**8**	UTSA-93	53.8	28	13
**9**	LIFM-100	21.0	13.4	14
**10**	BUT-11	53.5	31.5	15

Computational Details

Periodic density functional theory (DFT) calculations were performed using the CP2K package (version 2025.2)[16] within the Quickstep module, employing the Gaussian and plane-waves method.[17] The Perdew–Burke–Ernzerhof (PBE) exchange–correlation functional[18] was used together with Grimme's D3 dispersion correction[19] to account for long-range van der Waals interactions. Valence electrons were described with the molecularly optimized double-ζ polarized basis set (DZVP-MOLOPT-SR-GTH),[20] and core electrons were represented by Goedecker–Teter–Hutter (GTH) pseudopotentials.[21] The plane-wave density cutoff was set to 500 Ry with a relative cutoff of 50 Ry. Calculations were performed at the Γ-point with spin-polarized (unrestricted Kohn–Sham) treatment; the eight Cu(II) (d^9) centers were initialized in a ferromagnetic high-spin configuration (multiplicity = 9). The self-consistent field was converged to 10^{-6} Hartree. Gas-phase CO_2 and N_2 reference molecules were optimized in a 15 Å cubic box using the wavelet Poisson solver. All framework structures — the empty framework and the N_2- and CO_2-loaded frameworks — were fully relaxed, allowing both the atomic positions and the unit cell to vary (CELL_OPT), starting from the experimental lattices (Table S6). The DFT-optimized cells reproduce the experimental structures in both states, agreeing to within ~1% in volume. Crucially, DFT recovers the anisotropic, guest-induced breathing: the framework relaxes to the expanded cell when empty and to the compressed cell upon CO_2 loading, confirming that the moderate lattice flexibility is intrinsic to the CO_2–framework interaction.

Table S6. Fully Relaxed (DFT, CELL_OPT) and Experimental Lattice Parameters of the Empty and CO_2-loaded NCKU-56

Structure	***a*** **(Å)**	***b*** **(Å)**	***c*** **(Å)**	***β*** **(°)**	***V*** **(Å³)**
Empty framework, experimental	10.452	27.053	14.221	90.46	4021
Empty framework, DFT (CELL_OPT)	10.548	27.060	14.210	90.38	4056
CO_2-loaded framework, experimental	10.451	23.282	14.161	92.26	3443
CO_2-loaded framework, DFT (CELL_OPT)	10.549	23.688	13.830	92.72	3452

The adsorption energy per guest molecule was computed as

$$\Delta E_{ads} = [E_{(framework+n\ gas)} - E_{(framework)} - n\ E_{(gas)}] / n\ (n = 4),$$

where E(framework + n gas), E(framework), and E(gas) are the total energies of the guest-loaded framework, the empty framework, and the isolated gas molecule, respectively, and n = 4 is the number of guest molecules per unit cell. For CO_2, the guest positions were taken from the experimental CO_2-loaded structure, and E(framework + n gas) corresponds to the compressed CO_2-loaded cell, so ΔE_{ads} includes the energy of the guest-induced lattice contraction. For N_2, for which no guest-loaded structure is available experimentally, 12 initial configurations of four N_2

molecules were sampled within the channels, and the lowest-energy one was used; N_2 leaves the framework in its expanded form, so ΔE_{ads} reflects the host–guest interaction alone.

Computational Results

Across all sampled configurations, N_2 fails to localize at any specific binding site. It retains a free N–N geometry (1.12 Å) and forms only weak van der Waals contacts with the framework C–H groups (≈2.7–3.1 Å), remaining ≥ 4.2 Å from the Cu centers and showing no close contacts with the triazolate or quinone groups. CO_2, in contrast, localizes within the linker pocket (Figure 4B). It forms short, specific contacts with the triazolate rings through its electropositive carbon ($C(CO_2)\cdots N$ = 3.0–3.1 Å), consistent with the quadrupole–π interaction identified crystallographically, while remaining ~4.5 Å from the Cu centers and essentially linear (O–C–O ≥179°) — characteristic of weak, non-activating physisorption. This contrast — specific localization of CO_2 at the polar linker sites versus nonspecific physisorption of N_2 — provides an atomistic rationale for the selective CO_2 uptake. The calculated CO_2 adsorption energy (−33.6 kJ/mol per molecule, Table S7) is in good agreement with the experimental isosteric heat of adsorption (Q_{st} = 30.1 kJ/mol), both falling within the range characteristic of π-based physisorption (20–35 kJ/mol). The modest overestimation is expected, as the DFT value corresponds to the 0 K electronic limit and does not include zero-point or thermal corrections. N_2 binds more weakly (−23.9 kJ/mol), consistent with its nonspecific interaction with the framework; the ~10 kJ/mol difference in adsorption energy parallels the pronounced CO_2/N_2 selectivity observed experimentally.

Table S7. DFT-calculated Adsorption Energies and Key Host–Guest Contact Distances for CO_2 and N_2 in NCKU-56

Guest	ΔE_{ads} (kJ/mol)	Key host–guest contacts (Å)
CO_2	−33.6	$C(CO_2)\cdots N$(triazole) 3.02–3.07 (quadrupole–π)
N_2	−23.9	nonspecific; nearest C–H ≈2.7–3.1, ≥4.2 from Cu

References

(1) Oppenheim, J. J.; Ho, C.-H.; Alezi, D.; Andrews, J. L.; Chen, T.; Dinakar, B.; Paesani, F.; Dincă, M., Cooperative Interactions with Water Drive Hysteresis in a Hydrophilic Metal–Organic Framework. *Chem. Mater.* **2024**, *36*, 3395–3404.
(2) Porter, J. F.; McKay, G.; Choy, K. H., The prediction of sorption from a binary mixture of acidic dyes using single- and mixed-isotherm variants of the ideal adsorbed solute theory. *Chem. Eng. Sci.* **1999**, *54*, 5863−5885.
(3) Nuhnen, A.; Janiak, C., A practical guide to calculate the isosteric heat/enthalpy of adsorption via adsorption isotherms in metal–organic frameworks, MOFs. *Dalton Trans.* **2020**, *49*, 10295−10307.
(4) Wang, Y.-T.; Jalife, S.; Robles, A.; Đerić, M.; Wu, J. I.; Kaveevivitchai, W.; Miljanić, O. Š.; Chen, T.-H., Efficient CO_2/CO Separation by Pressure Swing Adsorption Using an Intrinsically Nanoporous Molecular Crystal. *ACS Appl. Nano Mater.* **2022**, *5*, 14021–14026.
(5) Gelb, L. D.; Gubbins, K. E., Characterization of Porous Glasses: Simulation Models, Adsorption Isotherms, and the Brunauer−Emmett−Teller Analysis Method. *Langmuir* **1998**, *14*, 2097–2111.
(6) Kim, K. C.; Yoon, T.-U.; Bae, Y.-S., Applicability of using CO_2 adsorption isotherms to determine BET surface areas of microporous materials. *Micropor. Mesopor. Mat.* **2016**, *224*, 294–301.
(7) Pal, A.; Chand, S.; Madden, D. G.; Franz, D.; Ritter, L.; Johnson, A.; Space, B.; Curtin, T.; Das, M. C., A Microporous Co-MOF for Highly Selective CO_2 Sorption in High Loadings Involving Aryl C–H···O═C═O Interactions: Combined Simulation and Breakthrough Studies. *Inorg. Chem.* **2019**, *58*, 11553–11560.
(8) Pal, A.; Chand, S.; Boquera, J. C.; Lloret, F.; Lin, J.-B.; Pal, S. C.; Das, M. C., Three Co(II) Metal–Organic Frameworks with Diverse Architectures for Selective Gas Sorption and Magnetic Studies. *Inorg. Chem.* **2019**, *58*, 6246–6256.
(9) Pal, A.; Chand, S.; Madden, D. G.; Franz, D.; Ritter, L.; Space, B.; Curtin, T.; Chand Pal, S.; Das, M. C., Immobilization of a Polar Sulfone Moiety onto the Pore Surface of a Humid-Stable MOF for Highly Efficient CO_2 Separation under Dry and Wet Environments through Direct CO_2–Sulfone Interactions. *ACS Appl. Mater. Interfaces* **2020**, *12*, 41177–41184.
(10) Chand, S.; Pal, A.; Das, M. C., A Moisture-Stable 3D Microporous CoII-Metal–Organic Framework with Potential for Highly Selective CO_2 Separation under Ambient Conditions. *Chem. Eur. J.* **2018**, *24*, 5982–5986.
(11) Sahoo, R.; Pramanik, B.; Krishna, R.; Das, M. C., A Chemically Robust 3D Interpenetrated MOF Toward One-Step Methane Purification Along with Six Other Multipurpose Gas Separations. *Small* **2026**, *22*, e72629.
(12) Song, C.; He, Y.; Li, B.; Ling, Y.; Wang, H.; Feng, Y.; Krishna, R.; Chen, B., Enhanced CO_2 sorption and selectivity by functionalization of a NbO-type metal–organic framework with

polarized benzothiadiazole moieties. *Chem. Commun.* **2014**, *50*, 12105–12108.
(13) Cui, H.; Ye, Y.; Liu, T.; Alothman, Z. A.; Alduhaish, O.; Lin, R.-B.; Chen, B., Isoreticular Microporous Metal–Organic Frameworks for Carbon Dioxide Capture. *Inorg. Chem.* **2020**, *59*, 17143–17148.
(14) Qiu, Q.-F.; Chen, C.-X.; Zeng, Z.; Wei, Z.-W.; Zhu, N.-X.; Cao, C.-C.; Wang, W.; Wang, D.; Wang, H.-P.; Jiang, J.-J., A Flexible–Robust Copper(II) Metal–Organic Framework Constructed from a Fluorinated Ligand for CO_2/R22 Capture. *Inorg. Chem.* **2020**, *59*, 14856–14860.
(15) Wang, B.; Huang, H.; Lv, X.-L.; Xie, Y.; Li, M.; Li, J.-R., Tuning CO_2 Selective Adsorption over N_2 and CH_4 in UiO-67 Analogues through Ligand Functionalization. *Inorg. Chem.* **2014**, *53*, 9254–9259.
(16) Kühne, T. D.; Iannuzzi, M.; Del Ben, M.; Rybkin, V. V.; Seewald, P.; Stein, F.; Laino, T.; Khaliullin, R. Z.; Schütt, O.; Schiffmann, F.; Golze, D.; Wilhelm, J.; Chulkov, S.; Bani-Hashemian, M. H.; Weber, V.; Borštnik, U.; Taillefumier, M.; Jakobovits, A. S.; Lazzaro, A.; Pabst, H.; Müller, T.; Schade, R.; Guidon, M.; Andermatt, S.; Holmberg, N.; Schenter, G. K.; Hehn, A.; Bussy, A.; Belleflamme, F.; Tabacchi, G.; Glöß, A.; Lass, M.; Bethune, I.; Mundy, C. J.; Plessl, C.; Watkins, M.; VandeVondele, J.; Krack, M.; Hutter, J., CP2K: An electronic structure and molecular dynamics software package - Quickstep: Efficient and accurate electronic structure calculations. *J. Chem. Phys.* **2020**, *152*, 194103.
(17) Lippert, G.; Hutter, J.; Parrinello, M., A hybrid Gaussian and plane wave density functional scheme. *Mol. Phys.* **1997**, *92*, 477–487.
(18) Perdew, J. P.; Burke, K.; Ernzerhof, M., Generalized Gradient Approximation Made Simple. *Phys. Rev. Lett.* **1996**, *77*, 3865–3868.
(19) Grimme, S.; Antony, J.; Ehrlich, S.; Krieg, H., A consistent and accurate ab initio parametrization of density functional dispersion correction (DFT-D) for the 94 elements H-Pu. *J. Chem. Phys.* **2010**, *132*, 154104.
(20) VandeVondele, J.; Hutter, J., Gaussian basis sets for accurate calculations on molecular systems in gas and condensed phases. *J. Chem. Phys.* **2007**, *127*, 114105.
(21) Goedecker, S.; Teter, M.; Hutter, J., Separable dual-space Gaussian pseudopotentials. *Phys. Rev. B.* **1996**, *54*, 1703–1710.
